# NK cells-derived extracellular vesicles potency in the B cell lymphoma biotherapy

**DOI:** 10.3389/fimmu.2024.1503857

**Published:** 2024-12-06

**Authors:** Serena Cecchetti, Cristina Federici, Rossella Canese, Egidio Iorio, Veronica Huber, Maria Elena Pisanu, Mattea Chirico, Elisabetta Iessi, Serena Camerini, Marialuisa Casella, Andrea Matteucci, Daniele Macchia, Massimo Spada, Luana Lugini

**Affiliations:** ^1^ Core Facilities, Confocal Microscopy Unit, Istituto Superiore di Sanità, Rome, Italy; ^2^ Department of Oncology and Molecular Medicine, Istituto Superiore di Sanità, Rome, Italy; ^3^ Core Facilities, MRI and HR-NMR Units, Istituto Superiore di Sanità, Rome, Italy; ^4^ Unit of Immunotherapy of human tumors, Istituto Nazionale dei Tumori, Milan, Italy; ^5^ Center for Gender-Specific Medicine, Istituto Superiore di Sanità, Rome, Italy; ^6^ Core Facilities, Mass Spectrometry Unit, Istituto Superiore di Sanità, Rome, Italy; ^7^ National Centre for Drug Research and Evaluation, Istituto Superiore di Sanità, Rome, Italy; ^8^ Centre for Animal Research and Welfare, Istituto Superiore di Sanità, Rome, Italy

**Keywords:** NK cells, extracellular vesicles, exosomes, microvesicles, B cell lymphoma, immunotherapy, biotherapy

## Abstract

**Introduction:**

Extracellular vesicles of Natural Killer cells (NKEV) exert an antitumor effect towards hematopoietic and solid tumors and have an immune modulating effect, suggesting a promising role in immune and biotherapy. In this study, a continuation of our former works, we demonstrated a network by mass spectrometry analysis between NKEV protein cargo and antitumor effects. Human healthy NKEV, both exosomes and microvesicles, have a significant and direct cytotoxic effect against human B cell lymphoma in *in vitro* and *in vivo* conditions.

**Methods:**

We isolated extracellular vesicles from *in vitro* amplified healthy human NK cells and their treatment efficacy was monitored by cytometry analyses, *in vivo* MRI/MRS measurements, ex vivo MRS analyses and immunohistochemistry.

**Results:**

We observed a remarkable NKEV cytotoxic effect, mainly by apoptosis, on B cell lymphoma *in vitro* when exosomes and microvesicles were administered simultaneously. *In vivo* results showed metabolic alterations in SCID mice xenografts after NKEV treatment, associated with a significant reduction of tumor growth (64%). In the *in vivo* ^1^H MR spectra we found a significant increase in the tumor lipid/lactate and in taurine signals, both considered as apotosis markers. Ex vivo lymphoma metabolomics revealed a significant increase in fatty acid (FA) pool and decrease in unsaturated and mono-unsaturated FA in treated groups, as compared to control one, thus suggesting an alteration of tumor homeostasis. Immunohistochemistry analyses confirmed the reduction of B-cell lymphoma proliferation rate, as well as the induction of apoptosis following the NKEV treatment.

**Conclusions:**

This study underscore the importance of NKEV as a novel biological acellular tool for B-cell lymphoma treatment, probably having a greater effect on combined treatment regimens. These nanovesicles have an extraordinary potential in innovative cancer immunotherapy, representing a safe and efficient tool naturally circulating in healthy individuals and ready to maintain the immune homeostasis, and therefore a good organism healthy state.

## Introduction

Lymphomas can develop in different organ in the body and are divided into Hodgkin’s lymphoma (about 10% of all lymphomas) and non-Hodgkin lymphoma (NHL) (1). NHL includes a various spectrum of cancers of the immune system, about 85–90% of it comes from B cells, whereas the remaining lymphomas derived from T cells or NK cells. Follicular lymphoma and diffuse large B-cell lymphoma (DLBCL), account for about 65% of all NHL (2, 3). In particular, large B-cell lymphomas, represent almost 30% of all cases of non-Hodgkin’s lymphoma. Patients typically present with progressive lymphadenopathy, extranodal disease, or both and require therapy. More than 60% of patients with aadvanced stage can be treated with R-CHOP (rituximab, cyclophosphamide, doxorubicin, vincristine, and prednisone) immune-chemotherapy. Patients with treatment failure after R-CHOP often have a poor outcome (3). Patients with relapsed refractory disease have significantly improved the outcomes with adoptive T-cell therapy (4).

NK cells (NK) belong to the innate immune system and are the first effective barrier of body defence from tumor cells (5). Individuals with low NK activity display an increased risk to develop cancer (6). Different mechanisms of tumor immune escape, such as the downregulation of MHC-I expression (7), the alteration of tumor microenvironment (TME) (8), and the secretion of tumor nanovesicles, generally block antitumor activity of immune cells, of NK cells in particular (9).

Little is known on the control of lymphomas by NK cells. NK cells and the NKG2D receptor play a role for control of lymphomas and that selection for NKG2D-L loss mutants provides a mechanism of tumor escape. In summary, the two-step process of NK-cell activation is a satisfactory hypothesis for explaining the interplay between NK cell functions and lymphoma growth. Following an initial cytotoxic activity of NK cells, several mechanisms seem to contribute to a progressive loss of NK-cell functions during the course of disease development. Critical role of the inhibitory (CD158a/b, NKG2A) and activatory (NKG2D, CD61) level on patients NK cells, are crucial in the B-cell lymphoma (10, 11). In recent years, the best results in cancer treatment have been obtained through the Immunotherapy (12). However, the percentage of patients that respond well to therapy is low (13).

Exosomes (EXO) and microvesicles (MV) are nanometer-sized secreted vesicles, 30-150 nm and 150-400 nm respectively, involved in numerous biological networks (14, 15). The *in vivo* immune regulatory properties and cell communication of these nanovesicles have been demonstrated in preclinical studies, supporting the possible use of exosomes in diagnosis and therapy of different disease, such as cancer (16). We have demonstrated for the first time that exosomes produced by NK cells isolated from blood of healthy donors (NKExo), display a potent cytotoxic activity against tumors *in vitro* (17). More recently we demonstrated that the fraction of tsg-101+/CD56+ exosomes in cancer patients was significantly lower respect to healthy donor and that two types of NK-derived extracellular vesicles (NKEV), exosomes and microvesicles, have immune modulatory properties, covering a promising role in the support of NK-mediated immunosurveillance (18). To date, other group of researchers demonstrated that NK cell extracellular vesicles of tumor origin or genetically modified NKEV exert an antitumor effect towards murine melanoma (19), neuroblastoma (20), glioblastoma (21), ovarian cancer (22).

In this study, we demonstrated that healthy NKEV both exosomes and microvesicles, have B cell lymphoma antitumor activity inducing apoptosis *in vitro* and *in vivo*. Moreover, the MRI/MRS analyses showed a significant increase in the tumor lipid lactate and taurine signals. These metabolic changes, considered as potential biomarkers of NKEV response, suggest that NKEV, by negatively affecting tumor homeostasis, are able to modify tumor lipidic metabolism, thus making the tumor more sensitive to combined therapies.

## Materials and methods

### NK cell expansion from human healthy donors

Buffy coats of al least 10 healthy donors (HD) were provided by Centro Trasfusionale Universitario Azienda Policlinico Umberto I in Rome, Italy (the study was approved by the ethical committee of Istituto Superiore di Sanita`, Rome, Italy, and donors gave written-informed consent to participate). Human PBMC were isolated from buffy coats by Ficoll-Histopaque 1077 gradient (Sigma-Aldrich, St. Louis, MO). Activated NK cells were, obtained starting from a coculture of PBMC (4 X 10^5^ cells/ml) with cobalt-irradiated (4000 rad) RPMI 8866 cells (B lymphoblastoid cell line) (1 X 10^5^ cells/ml) as described in detail in (17). The expanded NK cells subset was incubated with human rIL-2 (100 U/ml; Hoffman-La Roche, Nutley, NJ) for 3 days (cell viability > 90%). This culture method ensures an average of 25-fold increase in activated NK cell number, as well as more than 85-90% of NK cells population expressing CD16+ CD56+ molecules.

### Isolation of NK-derived extracellular vesicles

The culture supernatants of ex vivo expanded human NK cells were thawed at the time of experimental use and subjected to differential centrifugation as described in (18). Briefly, conditioned cell culture medium was centrifuged for 5 min at 300 × g and 20 min at 1,200 × g to remove cells and debris. NK-derived microvesicels (NKMVs) were pelleted for 30 min at 10,000 × g and washed in phosphate-buffered saline (PBS), while NK-derived exosomes (NKExo) were collected by ultracentrifugation at 100,000 × g for 90 min at 10°C using a Sorvall WX Ultra Series centrifuge in an F50L-2461.5 rotor (Thermo Scientific, Germany). The resulting pellet was first washed in PBS and then ultracentrifuged at 100,000 × g for 60 min. NK-derived extracellular vesicles (NKEv), that comprised both MVs and Exo, were resuspended in PBS, RPMI 1640 medium or phisiological solution depending on the experiments. The characterization of NKEV by Nanoparticle Tracking Analysis (NTA), scanning and transmission electron microscopy, and by proteomic analyses has been reported in our recent study on the immune modulating capability of human healthy donor-derived NKEV (18).

### Cell culture

SU-DHL-4, Human diffuse large follicular B-cell lymphoma cell line, (from ATCC) was cultured in RPMI 1640 medium (Life Technologies, Grand Island, NY), supplemented with 10% FBS, 100 U/ml penicillin, 100 mg/ml streptomycin (Life Technologies) and 2 mmol/l glutamine in a 5% CO2 environment at 37˚C. Cells were routinely tested for mycoplasma contamination by a PCR Mycoplasma detection kit (Venor GeM; Minerva Biolabs, Berlin, Germany).

### Bioinformatic analysis of proteomic data

Proteomic analysis was previously reported by our group (18). All the proteomics data have been deposited to the ProteomeXchange Consortium via the PRIDE partner repository with the dataset identifier PXD014894. The entire set of proteins identified in Exo and/or MV (3259 proteins in total) were analyzed by DAVID (23) to evaluate the enrichment in KEGG pathways. Only top 40 pathways with FDR <0.001 were taken into account.

### *In vitro* cell death evaluation

SU-DHL-4 cells were plated at 5 × 10^4^ cells per well in buffered RPMI medium. After 24 h, 10 µg Exo, 10 µg MVs or 20 µg EVs (Exo + MVs) were added to the cells. Following 24 h or 48 h of co-culture, cells were collected and washed twice with PBS1X. Apoptosis was evaluated by staining the cells with Annexin V-FITC and propidium iodide (PI) following manufacturer’s instructions (BioVision Incorporated, Milpitas, CA). After staining with Annexin V/PI for 15 min, cells were fixed in 1% PFA and analyzed on a Becton Dickinson FACScalibur, at least 10,000 cells per sample were analyzed using CellQuest software (Becton Dickinson Systems). All experiments were performed in triplicate, each being repeated at least three times. The mean fluorescence intensity (MFI) of samples was used in all cytotoxicity calculations. The maximum level of PI uptake was determined in target cells lysed by 0.5% Triton X-100. Cytotoxicity was expressed as the percentage of cell deaths among target cells: (number of dead cells/[number of dead cells + number of live cells]) x 100.

### Lymphoma xenograft animal model

All animals utilised in the present study were housed and treated in accordance with protocols approved by institutional authorities, in agreement with European Community Directives and with the Italian Law. All efforts were made to minimize animal suffering, to reduce the number of animals used and to adopt alternatives to *in vivo* testing whenever possible. The animals used in this experimentation were included in the research protocol “Exploitation of human NK cell-derived exosomes as cell-free support in tumor immunotherapy” approved by the experts from Service for Biotechnology and Animal Welfare and authorized bythe Italian Ministry of Health with the Decree nu 225/2016-PR of 1st March 2016.

CB.17 SCID/SCID female mice aged 4–5 weeks (Harlan, Milan, Italy) were kept under specific pathogen free conditions and fed ad libitum. Mice were injected subcutaneously in the right flank with 15 X 106 SU-DHL-4 human follicular B-cell lymphoma cells in 0.15 ml of saline solution (Baxter s.p.a.) and 0.15 ml of Matrigel (Corning^®^ Matrigel^®^ Growth Factor Reduced (GFR) Basement Membrane Matrix, Sigma-Aldrich). Mice were then randomly divided into three groups, of seven animals each. The Co-NKEV group was injected s.c. with 10 µg of NKEV simultaneously to the B Lymphoma cells injection, and then treated twice a week with an intratumoral injection of 10 µg of NKEV/mouse. The Post-NKEV group start to receive the NKEV treatment (10 µg) a week after the B Lymphoma cells injection, and treated twice a week with an intratumoral injection of 10 µg of NKEV/mouse, as the Co-NKEV group. The control group received an intratumoral injection of 0.2ml/mouse of saline solution twice a week. Tumor growth was estimated twice per week with caliper using the following formula (24):


tumor weight(mg)=12length(mm)Xwidth(mm)2=2

After 30 days from the injection of human B Lymphoma cells, all mice were sacrificed by cervical dislocation, following the guidelines of the Italian National Institute of Health. Tumors were analyzed with a two-way repeated measure ANOVA, with treatment (SAL, Co-NKEV, Post-NKEV) x time (7 points) design. To make tumor variances homogeneous at all time points, a square root transformation was applied to data. Time was a within subject factor, the treatment was a between subject factor. *Post hoc* analyses were conducted using Bonferroni test.

### *In vivo* MRI and MRS cytotoxicity evaluation

*In vivo* MR examinations were performed during treatment (from day 14 to day 24 after tumor implantation). MRI and MRS analyses were conducted at 4.7 T on a Varian/Agilent Inova horizontal bore system (Agilent, Palo Alto, USA) equipped with an actively shielded gradient coil (200 mT/m in 150 µs). In order to meet the requirements of spatial homogeneity and signal sensitivity, a volume coil was used for homogeneous transmission in combination with a receiver surface coil (RAPID Biomedical, Rimpar, Germany). MRI evaluation was performed by T1-weighted (T1W: TR/TE=600/18ms, thickness =0.8mm, FOV 20x20 mm2, matrix 256x128, 21 slices, 4 averages) and T2-weighted (T2W: TR/TE=3000/70ms, thickness =0.8mm, FOV 20x20 mm2, matrix 256x128, 21 slices, 4 averages) multislice spin echo MRI. Diffusion weighted MRI (DWI: TR/TE=2000/50 ms, thickness =1.2 mm, FOV 20x20 mm2, matrix 64x64, 12 slices, 2 averages and b-values= 0, 31, 69, 99, 200, 314, 707, 1105 s/mm2) was performed to measure the magnitude of diffusion of water molecules within tissue. This is performed by means of the apparent diffusion coefficient (ADC) parameter that is calculated by using the mono-exponential decay of signals for b-values over 100 s/mm2. Water motion in the capillary network (perfusion) influence the DWI signal. To separate perfusion- and diffusion-related effects can be used the intra voxel incoherent motion (IVIM) model by assuming biexponential behaviour of signal decay. IVIM model can estimate the true diffusion coefficient (D) and perfusion-related coefficient (D*) and perfusion fraction (f). Changes in ADC and in IVIM parameters may be useful for early tumor response assessment as observed in animal and human studies (25, 26). We perform both monoexponential fit for b values over 100 s/mm2 to estimate the ADC and the biexponential IVIM analysis to measure perfusion related parameters. In order to study the biologic heterogeneity of tumor by classifying domains of different diffusivity, which may have prognostic and predictive implication (27), in addition to the estimation of the average ADC, we performed histogram analysis of ADC values. ADCmean, ADCmedian, kurtosis and skewness were determined from ADC histogram analyses. Kurtosis measures how sharp is the peak relative to a standard bell curve: sharp peaks indicate homogeneous tumors. Skewness indicates the departure from horizontal symmetry, which suggests the presence of areas of higher ADC (indicative of necrosis) or lower ADC (proliferating areas) within the tumor. Histogram analyses with their related parameters have been also performed for b value less than 200 s/mm2 i.e. for fast diffusing spins (ADCperfusionmean; ADCperfusionmedian, kurtosisperfusion and skewnessperfusion) (28). All the MR-related parameters (i.e. detectable metabolites, T2, D, D*, f, ADCmean, ADCmedian, kurtosis, skewness) were evaluated with two-tailed t-test and multivariate analysis. *Post hoc* analyses were conducted using Bonferroni test.

The quantitative *in vivo* MRS protocol (PRESS, TR/TE = 4000/23 ms) included the T2-corrected water signal as internal reference and the LCModel fitting routine (29). Fourteen metabolites (selected on the basis of *in vitro* analyses of aqueous tissue extracts of these tumors) were included in the basis set: alanine (Ala), creatine (Cr), phosphocreatine (PCr), glycine (Glyc), glucose (Glc), glutamate (Glu), glutamine (Gln), glutathione (GS), glycerophosphocholine (GPC), phosphocholine (PCho), myo-inositol (m-Ins), lactate (Lac), scyllo-inositol (Scyllo-Ins), taurine (Tau). Spectra of lipids (Lip) and macromolecules were also included in the basis set as detected in our previous experience in other tumor types (29). Both GABA and NAA metabolites have not been usually observed in lymphomas (30), so we excluded their basis from the basis set utilized by LCModel for our quantitative analyses. We included in the analysis only those metabolites that were estimated to have Cramer-Rao lower bounds (CRLB) less than 20% (which corresponded to an estimated concentration error <0.2 µmol/g).

### Extraction of tissue aqueous and organic metabolites for metabolomics analysis by HR-NMR

Deuterated reagents (methanol (CD3OD), chloroform (CDCl3)) and deuterium oxide (D2O) (Cambridge Isotope Laboratories, Inc.) and 3-(trimethylsilyl) propionic-2,2,3,3-d4 acid sodium salt (TSP) (Merck & Co, Montreal, Canada) were used for extraction of aqueous and organic metabolites. All samples were stored at −80°C until metabolomics analysis by NMR spectroscopy. All frozen tissues pieces were weighted and placed into pre-cooled (dry ice) homogenization tubes. Ice-cold extraction solvent (methanol/chloroform/water (1:1:1)) was added to each tube and the tissues were then homogenized by homogenizator (Ika Homogenizer T10, Sigma-Aldrich, Milan, Italy) three times over 30s with 30s pause intervals to ensure constant temperatures during homogenization. At least 24h after, polar and lipid phases, containing water soluble and organic cellular metabolites respectively, were separated by centrifugation at 20,000 x g at 4°C for 30 min. Afterward the polar methanol/water phase was lyophilized by using a rotary evaporator (Savant RTV 4104 freeze dryer), while the organic lipid phase was collected in tube and chloroform evaporated under nitrogen gas flow and residues stored at -20°C. The aqueous and lipid fractions were then reconstituted in 700 µl D2O using TSP (0.1mM) as NMR internal standards or suspended in a CD3OD/CDCl3 solution (2:1 v/v) with internal control tetramethylsilane (TMS, 0.05%), respectively.

### Metabolic analysis by HR-NMR spectroscopy

High-resolution 1H-NMR analysis was performed at 25°C at 400 MHz (Bruker AVANCE spectrometer, Karlsruhe, Germany) on aqueous and organic extracts using a 60°flip angle pulse, preceded by 2s presaturation for water signal suppression (interpulse delay 2s, acquisition time 1.70s, spectral width 12 ppm, 16,000 data points, 320 scans) as previously described (28). Free induction decays were zero-filled to 32,000 data points and Fourier-transformed; a cubic splines model function was applied for baseline correction. Metabolites were identified according to our previous studies (by spiking with metabolite standards) and the databases published on the Human Metabolome Database site (http://www.hmdb.ca). The absolute quantification of metabolites was determined by comparing the integral of each metabolite to the integral of reference standard TSP, and corrected by respective proton numbers for metabolite and TS at equilibrium of magnetization. Metabolite quantification was expressed as nanomoles/g tissue and then converted into metabolite percentage (relative to total metabolites evaluated in each sample) to enable comparisons among the samples. The relative quantification in organic fraction of metabolites was determined by comparing the integral of each metabolite to the total metabolites evaluated in each sample and expressed as percentage relative to total metabolites.

### Immunohystochemistry and confocal laser scanning microscopy

A portion of the resected tumors was processed for immunofluorescence analyses. In particular, tumors specimens were fixed with PFA 4% and 0.12M sucrose in PBS, for 24 h at 4°C. After fixation, samples were rinsed three times with 5% sucrose/0.15mM CaCl2 in PBS for 10 min each, and then dehydrated overnight with 30% sucrose/0.15mM CaCl2; thus, samples were frozen at -80°C. Frozen tumor specimens were then mounted in Tissue-Tek^®^ O.C.T. Compound (Sakura, Gentaur, Kampenhout, Belgium), serial 10 μm-thick sections were cut using a Leica CM 1860 cryostat (Leica Biosystems, Buccinasco, MI, Italy), and mounted on positive charged microscope slides (SuperFrost Plus, Menzel Glaser, Thermo Fisher Scientific, Rodano, MI, Italy). Sections were incubated in 1% horse serum in PBS for 30 minutes at room temperature and then overnight with the following primary antibodies: CD19 (1:50 BD Biosciences, Milan, Italy), CD38 (1:50, Immunological Sciences, Rome, Italy), Ki67 (1:100, BD Biosciences, Milan, Italy), Bax (1:100, BD Biosciences, Milan, Italy), Bcl2 (1:50, Dako, Agilent Technologies Italia, Cernusco sul Naviglio, MI, Italy). Slides were washed 3 times with PBS and then incubated 2h at room temperature with the appropriated Alexa Fluor^®^ fluorochrome-conjugated secondary antibodies. For the TUNEL assay the DeadEnd™ Fluorometric TUNEL System, (Promega, Milan, Italy) was used according to the manufacturer’s instructions. DAPI solution (300 nM in PBS; Sigma-Aldrich, Merk Life Science, Milan, Italy) was added to each slide and incubated 10 minutes at room temperature. After this step slides were rinsed with PBS and mounted with Vectashield mounting Medium (Vector laboratories, Burlingame, CA, United States). Images were captured with a Zeiss LSM980 apparatus (Zeiss, Oberkochen, Germany), equipped with a Pln Apo 40x/1.4 oil objective, 0.55NA, Airyscan2 and excitation spectral laser lines at 405, 488, 543, 633 nm. Image acquisition and processing were carried out using Zen Blue edition 3.3 (Zeiss). CLSM images were obtained by Z-projection with up to 25–30 serial sections of 0.15-0.20 µm thick, taken from the bottom to the edge of the tissue sections, several fields were analyzed for each condition and representative results are shown. Quantitative analyses were performed using the Zen 3.3 software and mean fluorescence intensity (MFI) ± SEM of signals were determined in at least 6-7 independent experiment per marker (≥300 cells per experimental condition, in each repeat), and plotted following the formula: Final MFI = MFI (region of interest) - MFI (background).

### Statistical analysis

Statistical analyses were performed by paired and unpaired Student’s t-test, ANOVA one way or two way test for significant differences between groups, and by *post hoc* analyses using Bonferroni or Tukey’s, as indicated. Data are expressed as mean ± SEM or mean ± SD, as indicated, using GraphPad Prism 5, and p<0.05 or less were considered to be significant.

### Ethics statement

The studies involving human participants were reviewed and approved by the Ethical Committee of Azienda Policlinico Umberto I, University Sapienza, Rome, Italy. The patients/participants provided their written informed consent to participate in this study.

## Results

### Isolation of NK-derived extracellular vesicles containing both NK-derived exosomes and NK-derived microvesicles

Activated NK cells were obtained starting from a co-culture of healthy donors PBMC with cobalt-irradiated B lymphoblastoid cell line (RPMI 8866 cells) at the ratio 1:4, as described in detail in (17). After a 10-day expansion phase of this human healthy NK cells subset, human rIL-2 was added to the cell culture and incubated for additional 3 days (cell viability > 90%). At the end of the culture, supernatants were subjected to differential centrifugation, as previously described in (17, 18).

NK-derived extracellular vesicles (NKEV) were isolated by centrifugation at 100,000 x g, while NK-derived microvesicles (NKMVs) and NK-derived exosomes (NKExo) were obtained by centrifugation at 10,000 x g and 100,000 x g, respectively. All these NKEV were extensively characterized, as reported in our previous work (18).

**Figure 1:**
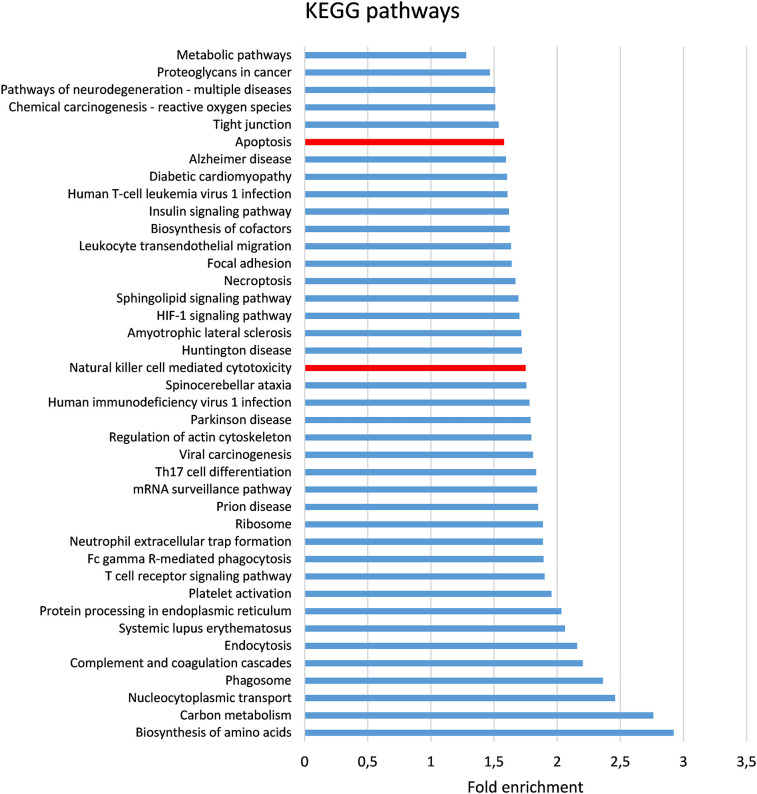
Bioinformatic analysis of proteomic data. The entire set of proteins previously (18) identified in Exo and/or MV (3259 proteins in total) was analyzed by DAVID (23) to evaluate the enrichment in KEGG pathways. The pathways (FDR < 0.001) are shown in order of their fold enrichment. Bars relative to Apoptosis and Natural cell mediated cytotoxicity pathways are in red.

**Figure 2:**
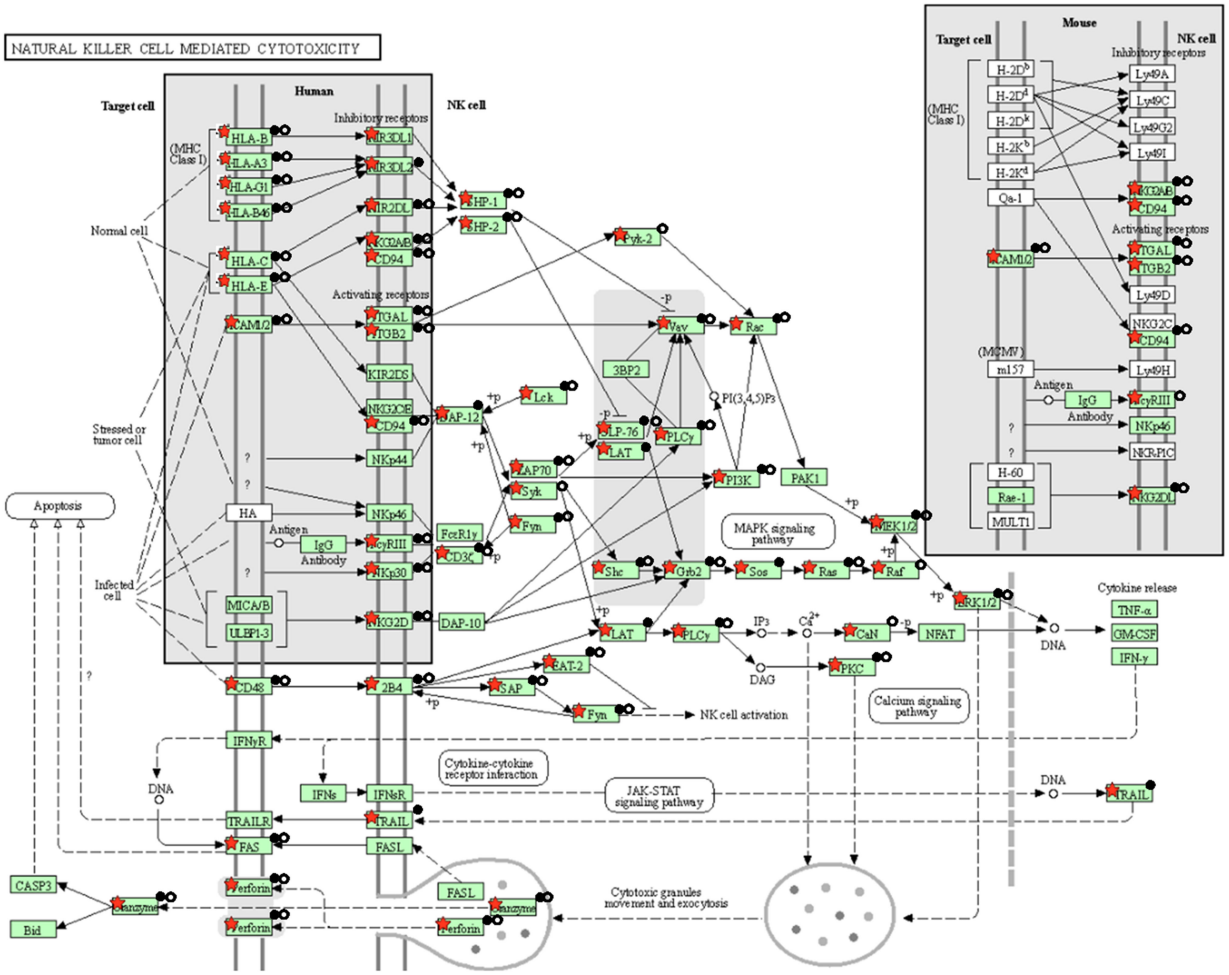
KEGG natural cell mediated cytotoxicity pathway. The KEGG natural cell mediated cytotoxicity pathway is shown and the red stars indicate 59 proteins identified in the Exo and/or MV dataset. Solid and empty circles indicate proteins detected in Exo an MV preparation, respectively.

**Figure 3:**
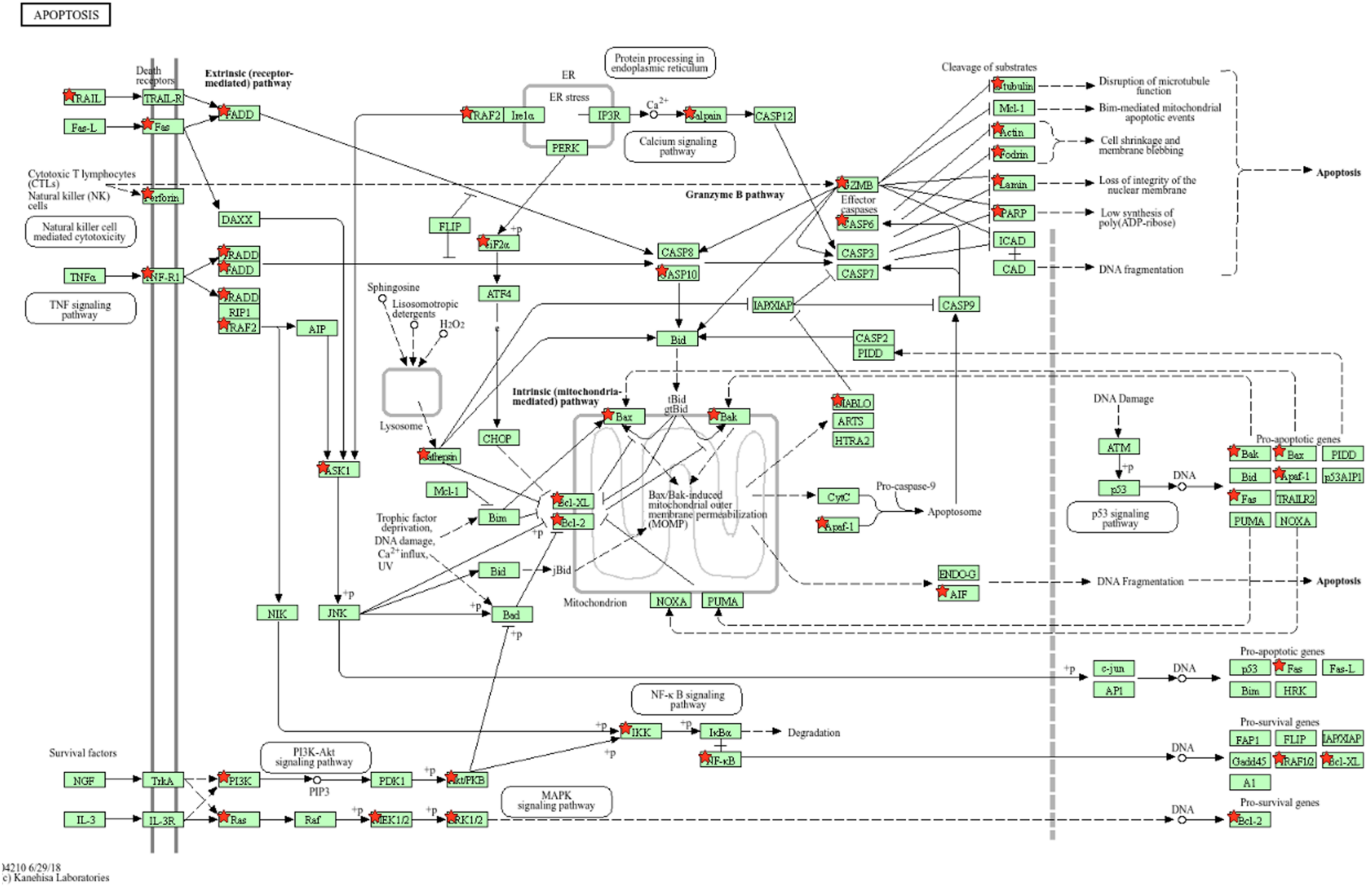
KEGG apoptosis pathway. The KEGG apoptosis pathway is shown and the red stars indicate 59 proteins identified in the Exo and/or MV dataset. Solid and empty circles indicate proteins detected in Exo and MV preparation, respectively.

The present study is an extension of our previous works (17, 18), we herein investigated the capability of human healthy NKEV in reducing viability and inducing cytotoxicity in cancer cells, both *in vitro* and *in vivo* in a B lymphoma model. The entire set of proteins identified in Exo and/or MV (3259 proteins in total) and reported in Federici et al. (18) was analyzed by DAVID to evaluate a possible enrichment in KEGG pathways. As shown in **Figure 1**, several pathways were found enriched. In particular, we focused our attention in NK cell mediated cytotoxicity and apoptosis pathways (red bars in **Figure 1**), that are described in the cartoons in **Figures 2**, **3**, respectively. Many proteins involved in these two pathways have been identified in our set (red stars in **Figures 2**, **3**, see also **Supplementary Tables 1**, **2**), and most of them were detected both in Exo (solid circles **Figures 2**, **3**) and MV (empty circles **Figures 2**, **3**). In the KEGG pathway enrichment analysis performed by using DAVID we also found the necroptosis, the Neutrophil Extracellular Trap (NET) and the biosynthesis of amino acids pathways (**Supplementary Figures 1**–**3**). This functional enrichment observed in the NKEV protein composition suggest that they could exert a cytotoxic effect and that their presence in plasma could play a key role to support the efficiency of the immune system.

### Cytotoxic effects of NKEV on B lymphoma cells *in vitro*

**Figure 4:**
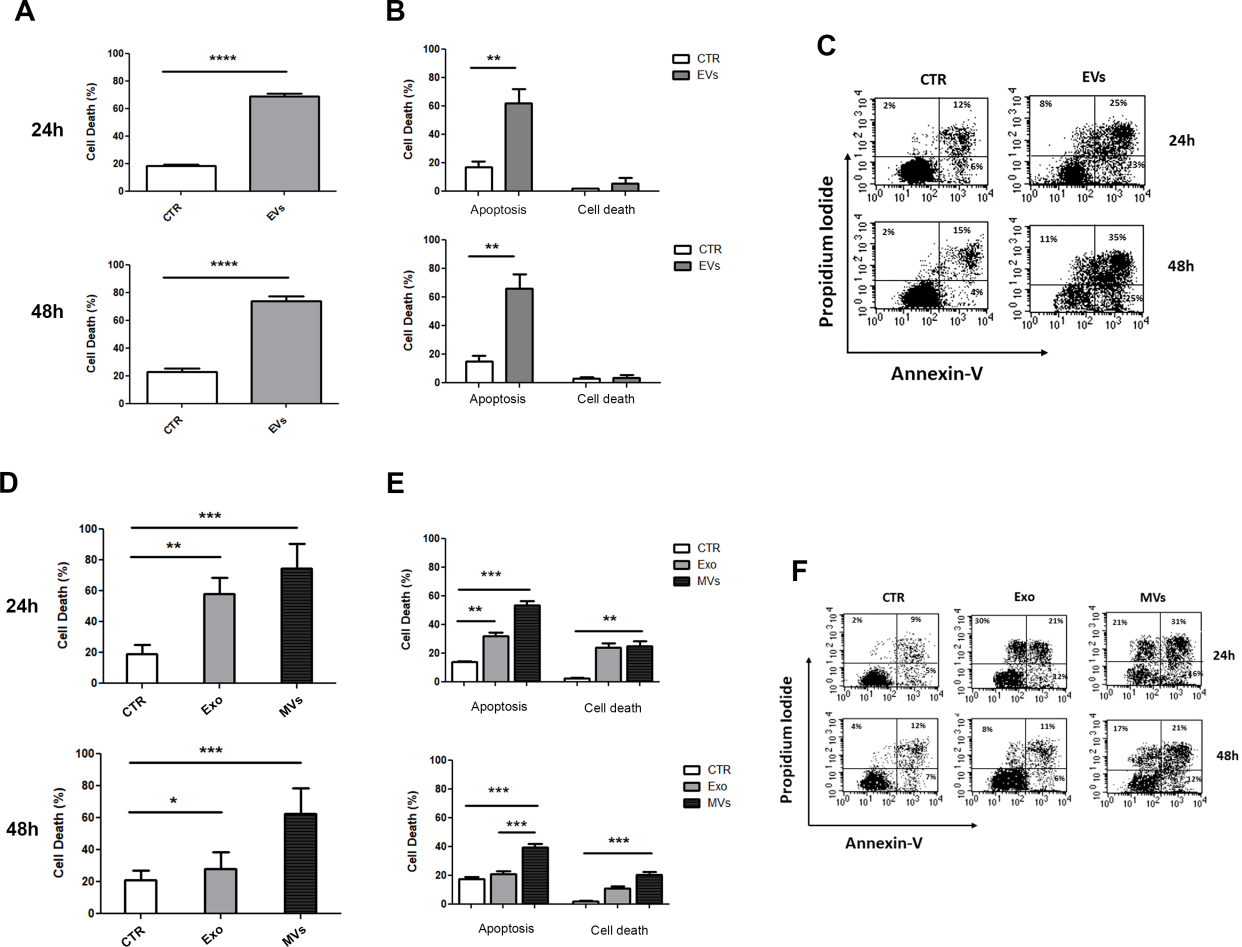
*In vitro* effects of NKEV on B lymphoma cells’ viability. **(A)** Flow cytometry analyses of SU-DHL-4 cells incubated with NK-derived extracellular vesicles (EVs) for 24h and 48h or left untreated (CTR). **(B)** Cytotoxicity was evaluated for 24h and 48h using the AnnexinV-FITC/PI dual staining assay, and it was expressed as the percentage of apoptosis and other cell death among target cells: (number of dead cells/ [number of dead cells + number of live cells]) x 100. **(C)** Representative dot plots of data shown in **(B)**. **(D)** Flow cytometry analyses of SU-DHL-4 cells incubated with NK-derived exosomes (Exo) and microvesicles (MVs) for 24h and 48h or left untreated (CTR). **(E)** Cytotoxicity was evaluated for 24h and 48h using the AnnexinV-FITC/PI dual staining assay, and it was expressed as the percentage of apoptosis and other cell death among target cells: (number of dead cells/ [number of dead cells + number of live cells]) x 100. **(F)** Representative dot plots of data shown in **(E)**. *p<0.05; **p<0.01; ***p<0.001; p<0.0001.

In order to evaluate the cytotoxic effects exerted by NKEV, NKExo and NKMVs on tumor cell viability we performed flow cytometry analyses on SU-DHL-4 B lymphoma cells after incubation with NKEV for 24h and 48h. In particular, cytotoxicity was evaluated using the AnnexinV-FITC/PI dual staining assay (**Figure 4**). As reported, NKEV were able to induce up to 60% of cell death, mostly by apoptosis, both after 24h and 48h of co-culture with SU-DHL-4 cells (**Figures 4A, B**). Representative cytometry dot plots are shown in **Figure 4C**. We also investigated the single contribution of NKExo and NKMVs in inducing tumor cell death (**Figures 4D, E**). We observed that NKMVs are able to induce at least 50% and 40% of cell death by apoptosis after 24h and 48h, respectively (**Figure 4E**). Whereas, NKExo induced up to 30% of apoptosis after 24h and 20% after 48h of co-culture (**Figure 4E**). Representative cytometry dot plots are shown in **Figure 4F**. Moreover, non-apoptotic cell death remained at low levels during NKEV incubation (less than 10% at both 24h and 48h, **Figure 4B**). Besides, co-culture with NKExo and NKMVs increased non-apoptotic cell death, up to 30% after 24h of incubation with both kind of nanovesicles, while we observed a slight reduction after 48h (up to 10% with NKExo and 20% with NKMVs) (**Figure 4E**).

### Effects of NK-derived extracellular vesicles on xenograft tumor growth *in vivo*

**Figure 5:**
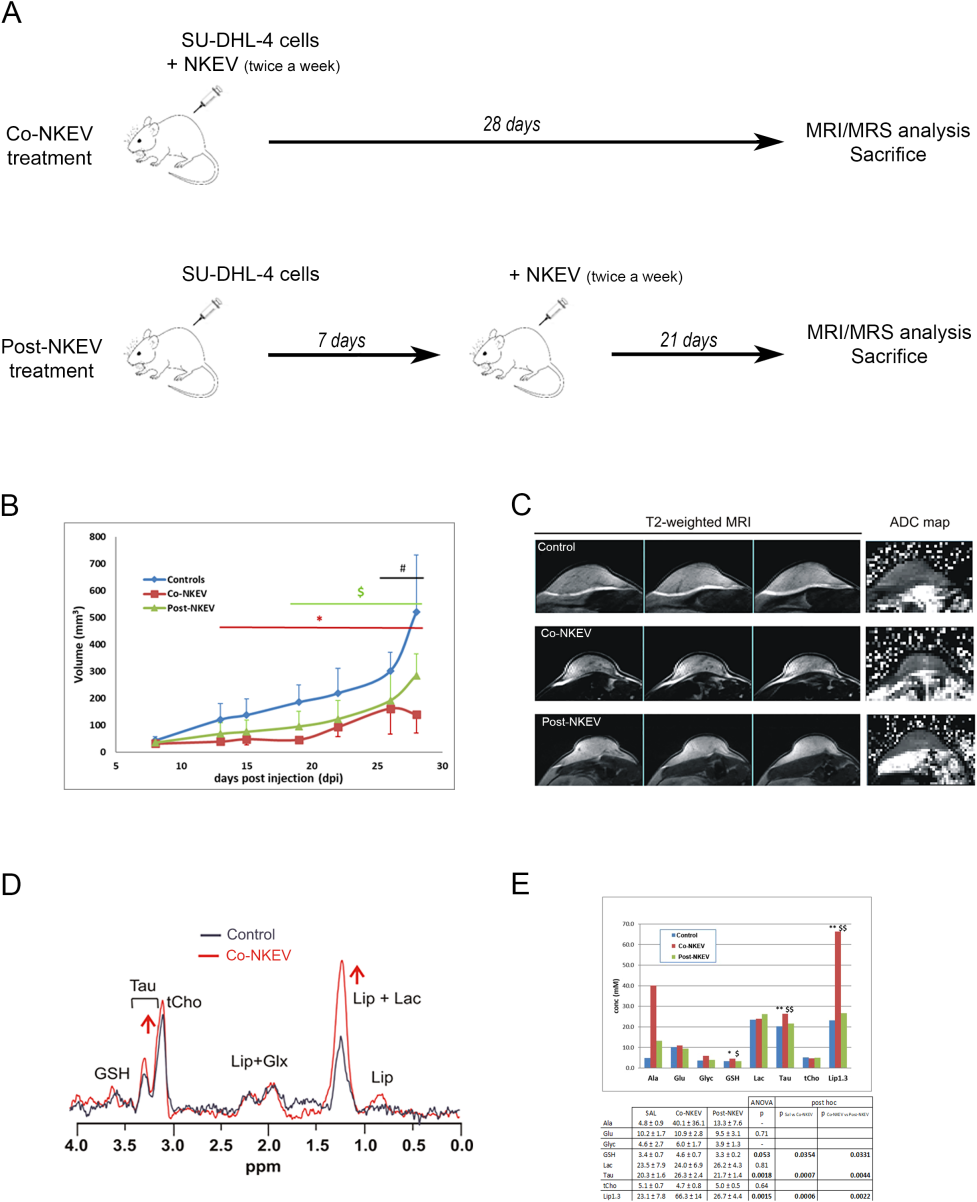
*In vivo* effects of NKEV on SU-DHL-4 tumor xenografts. **(A)** Scheme of the animal experiment protocol in the present study. All the animals were sacrificed at the end of 28 days. **(B)** Evaluation of tumor growth of SU-DHL-4 tumor xenografts by caliper. The Co-NKEV group (red line) was injected with NKEV simultaneously to the B-cells lymphoma injection, and then treated twice a week with an intratumoral injection of NKEV/mouse. The Post-NKEV group (green line) start to receive the NKEV treatment a week after the B-cells lymphoma injection, and treated twice a week with an intratumoral injection of NKEV/mouse, as the Co-NKEV group. The control group received an intratumoral injection of saline solution twice a week. * indicates posthoc significance between controls and Co-NKEV (p<0.05); $ indicates significance between controls and Post-NKEV (p<0.05); # indicates significance between Co-NKEV and Post-NKEV (p<0.05). **(C)** Representative examples of three consecutive coronal slices of T2‐weighted MRI of control, Co-NKEV and Post-NKEV treated xenograft (left panel) and the corresponding ADC map (right panel) acquired between 20 and 30 dpi. **(D)** 1H MR spectra acquired *in vivo* from a control and a Co-NKEV xenograft. Peak assignment: GSH, glutathione; Tau, taurine; tCho, choline containing compounds; Lip, lipids; GLX, glutamine plus glutamate; Lac, lactate. **(E)** Absolute quantification (mM) of *in vivo* MRS visible metabolites in SU-DHL-4 xenografts in saline treated tumors (control), in Co- and Post-NKEV-treated tumors. Error bars = ± SD (n=7). In the table below is reported ANOVA statistical analysis and posthoc significance among the groups of the analyzed metabolites. Asterisks (*) indicate posthoc significance between controls and Co-NKEV; $ indicate significance between controls and Post-NKEV. *P<0.05 CTR vs Co-NKEV; **P<0.01 CTR vs Co-NKEV; $ P<0.05 CTR vs Post-NKEV; $$ P<0.01 Co-NKEV vs Post-NKEV; # P<0.05 Co-NKEV vs Post-NKEV. Ala, alanine; Glyc, glycine; GSH, glutathione; Glu, glutamate; Lac, lactic acid; Lip, lipid signal at 1.3 pppm; tCho, choline containing metabolites; Tau, taurine.

In the light of the results obtained with NKEV *in vitro*, mouse xenograft models of SU-DHL-4 cells were used to determine *in vivo* effects of NKEV in B lymphoma tumors (**Figure 5A**). SU-DHL-4 cells were subcutaneously inoculated in SCID immunodeficient mice, afterwards randomly divided into three groups (n=7 per group). One group of mice (the Co-NKEV group) received NKEV (10 µg) as a co-injection with tumor cells, and then these mice were treated twice a week with an intratumoral injection of 10 µg of NKEV/mouse (**Figure 5A**). The Post-NKEV group, instead, started to receive the NKEV treatment (10 µg) 7 days after SU-DHL-4 cells injection, and treated twice a week with an intratumoral injection of 10 µg of NKEV/mouse, as for the Co-NKEV group (**Figure 5A**). An intratumoral injection of 0.2 ml of saline solution/mouse was administered twice a week to the control group. Tumor growth was monitored twice per week during the follow-up of treatment by standard external caliper measurements, until animal sacrifice.

NKEV-treated mice displayed a significant reduction in tumor growth at all-time points, starting from day 13 after the injection for the Co-NKEV group (simultaneous inoculation of B lymphoma cells with NKEV, **Figure 5B**, red line), and day 19 for the Post-NK group (NKEV treatment a week after the B lymphoma cells injection, **Figure 5B**, green line), as compared to control group (**Figure 5B**, blue line). The antitumor effects exerted by NKEV were more pronounced in the Co-NKEV group (-64% tumor growth respect to control group at day 13 after the injection), compared to the Post-NKEV group (-44% tumor growth respect to control group at day 19 after the injection) [ANOVA repeated measurement, with treatment (saline, Co-NKEV and Post-NKEV) x time (7 points) design, p=0.003, Bonferroni *post hoc* analyses]. Moreover, we also abserved differences in the engraftment among the groups. After 10 days from inoculation, in the Co-NKEV group the tumor mass had grown 27% less than control group and 11% less than the Post-NKEV group.

### 4 *In vivo* magnetic resonance imaging and spectroscopy on SU-DHL-xenograft models

Next, we wanted to highlight the differences in the texture inside the tumor more than volume reduction, so we performed *in vivo* MRI of all xenograft tumor-bearing mice. The analyses showed homogeneous intensity in both T1 and T2, without any significant morphological alteration. T2-weighted images and the ADC maps of representative tumors are shown in **Figure 5C**. One-way ANOVA did not detect any significant changes in the T2 (measured by MRS) and in the diffusivity parameters derived from DWI, neither with IVIM method nor with the monoexponential model. Data (mean ± SD) and the corresponding level of significance (p) resulting from the ANOVA analyses are summarised in **Table 1**. The homogenous tumor allowed the acquisition of good quality spectra. Examples of spectra obtained from a control and a Co-NKEV-treated xenograft are shown in **Figure 5D**. The *in vivo* quantification (mM) of the MR visible metabolites in the three group of xenografts showed a significant increase in the tumor lipid/lactate signal at 1.33 ppm (one-way ANOVA, p=0.0015) and in taurine signal (one-way ANOVA, p=0.0018) in the Co-NKEV group, compared to control and to the Post-NKEV group, which could be attributed to increased cell apoptosis (31, 32) (**Figure 5E**). A trend of increase of glutathione in the Co-NKEV group with respect to control and to the Post-NKEV group was also found (**Figure 5E**).

**Table 1:** T2 and diffusivity parameters derived from DWI.

MRI parameters		SAL	Co-NKEV	Post-NKEV	p
T2 (ms)		63 ± 1	61 ± 4	62 ± 2	0.34
IVIM analysis	D (x 10^-4^ mm^2^/s)	3.2 ± 0.2	3.0 ± 0.6	3.5 ± 0.4	0.24
	D*(x10^-4^ mm^2^/s)	15 ± 9	21 ± 6	15 ± 10	0.69
	f	9 ± 3 x 10^-2^	10 ± 8 x 10^-2^	12 ± 8 x 10^-2^	0.86
Histogram analysis from mono-exponential model	ADC_mean_ (x 10^-4^ mm^2^/s)	3.8 ± 0.5	3.6 ± 0.2	3.7 ± 0.3	0.92
	ADC_median_ (x 10^-4^ mm^2^/s)	3.6 ± 0.4	3.5 ± 0.2	3.6 ± 0.3	0.90
	Skewness	2.1 ± 1.7	1.5 ± 1.5	0.7 ± 0.8	0.52
	Kurtosis	5.8 ± 3.2	12 ± 10	2.3 ± 0.5	0.15

### Metabolomic profile of SU-DHL-4 xenograft models by proton nuclear magnetic resonance

**Figure 6:**
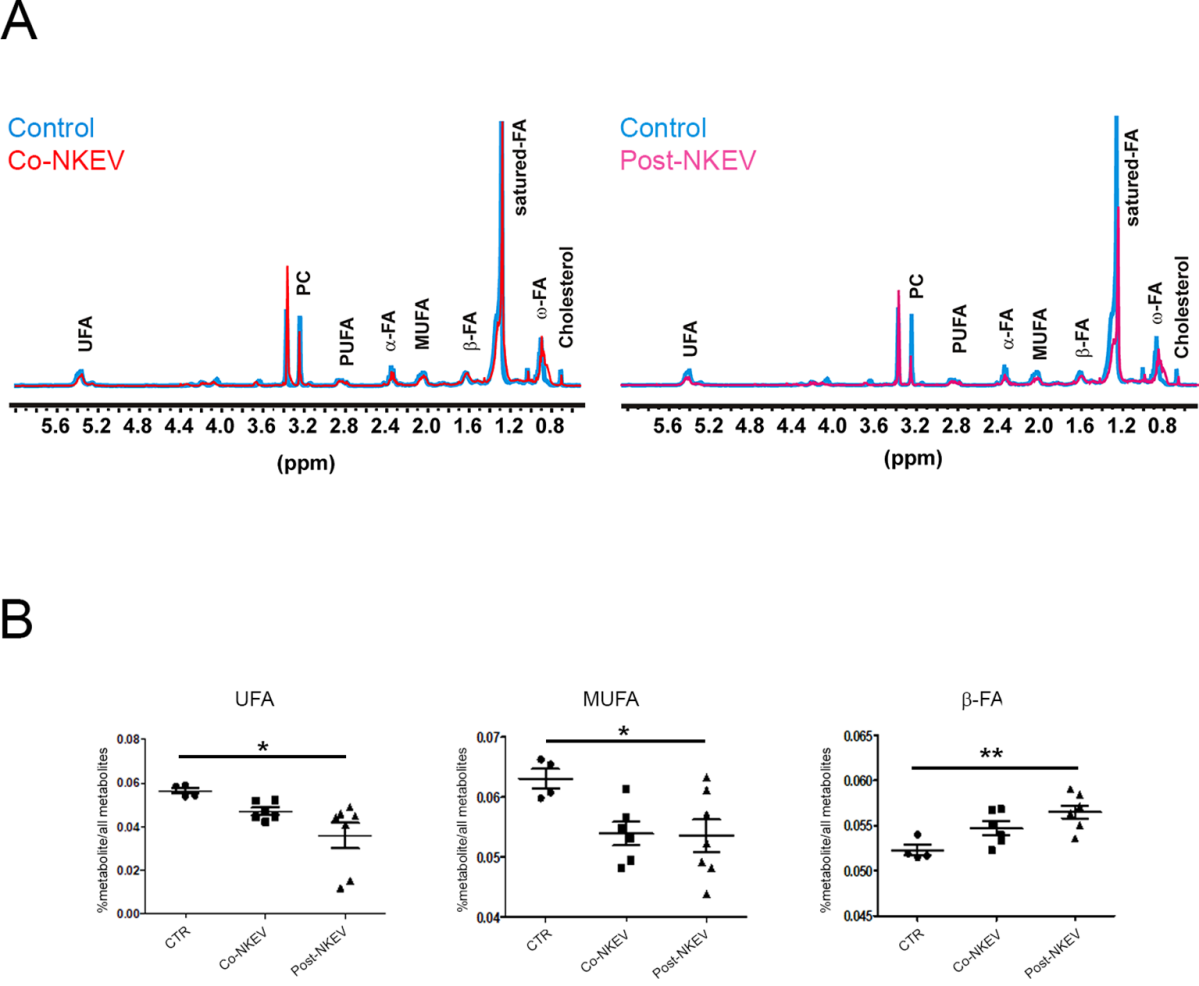
Metabolomic profile of SU-DHL-4 xenograft models by 1H-NMR. **(A)** Representative 1H HR -NMR spectra (9.4T) of lipid fractions of control tissue extracts compared with Co-NKEV (on the left) and Post-NKEV tissue extracts (on the right). Peak assignment: signals of fat acid (FA) determined at 0.9 ppm (ω-CH3 of FA), at 1.56 ppm (-CH2-COO of FA; α) and at 2.40 ppm (-CH2-CH2-COO of FA; β); monounsaturated fat acids (MUFA), polyunsaturated fat acids (PUFA); pool of phosphatidylcholine (PC) plus Lyso-PC and Triacylglicerids (TAG). **(B)** Scatter plot represent the relative quantification of lipid profiles obtained from organic tissue extracts in Post-NKEV, Co-NKEV and control tissues. Statistical analyses determined by ANOVA one way test: UFA, unsaturated fatty acid (p=0.02, R square 0,419); MUFA monounsaturated fatty acid (p=0.04 R square 0,369); CH2-CH2-COO-(β-FA) (p=0.01, R square 0,523). p value is referred to significant differences between groups. *p<0.05; **p<0.01.

In order to better characterize the intratumoral metabolomics profile and to define the metabolic effects of NKEV treatment on B-cell lymphoma xenograft model, we performed an ex vivo analysis by using high resolution (HR)-NMR approach which offers quantitative determination of aqueous and lipid metabolites linked to different biological pathways. First, we measured the organic fraction of tissue extracts and we found significant changes in the pool of fat acid (FA) and in their degree of unsaturation (UFA). In particular, we observed a significant (p ≤ 0.05) increase in FA pool and decrease in UFA and mono-unsaturation (MUFA) in both Co-NKEV group (n=6) and in Post-NKEV group (n=7) as compared to control group (**Figure 6**; **Supplementary Table 3**). In parallel, the content of fraction of saturated FA was significantly different in both treated groups as well (p ≤ 0.05). No differences were found in the levels of triacylglycerols (TAG), phospholipids, pool of phosphatidylcholine (PC) plus Lyso-PC and total cholesterol between both treated groups and control tumors. This active FA metabolism found in organic fraction following both treatments could be supported by average increase (although not significant) in some metabolites linked to branched aminoacids (valine and isoleucine), succinic acid and acetic acid found in aqueous fraction of tissues extracts of both Co- NKEV and Post-NKEV compared to control tissue extracts (**Supplementary Table 4**).

### Immunohistochemistry and confocal microscopy analyses of SU-DHL-4 xenografts

**Figure 7:**
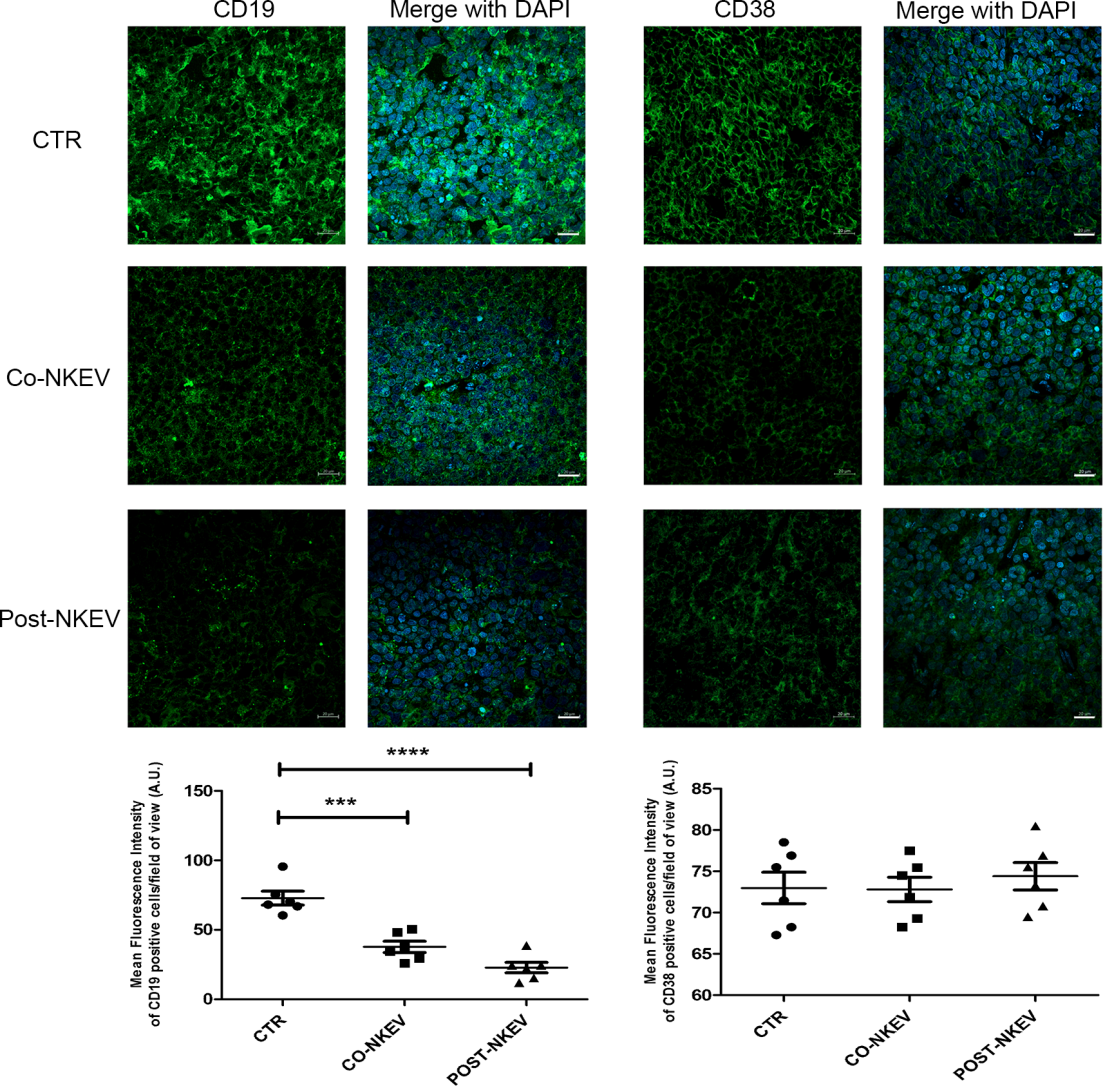
Ex vivo fluorescence analyses of SU-DHL-4 xenografts. Frozen tumor tissues with 10 µm thickness derived from control, Co-NKEV and Post-NKEV groups were stained using anti CD19 and CD38 Abs followed by the respective Alexa Fluor 488-conjugated secondary Abs and then analyzed by Confocal Laser Scanning Microscopy. CLSM analyses were performed on at least 4 tumor xenografts/group and representative examples are shown. Below each panel, the relative quantification analysis of mean fluorescence intensity is reported. MFI ± SEM of CD19 and CD38 signal per field of view (≥300 cells per experimental condition, in 6 independent experiments per marker), and plotted in the corresponding graphs, defining final MFI as MFI (region of interest [ROI]) - MFI (background). Bars indicate mean ± SEM, p values were calculated by one-way ANOVA with Tukey’s correction for multiple testing. ***p<0.001; ****p<0.0001.

**Figure 8:**
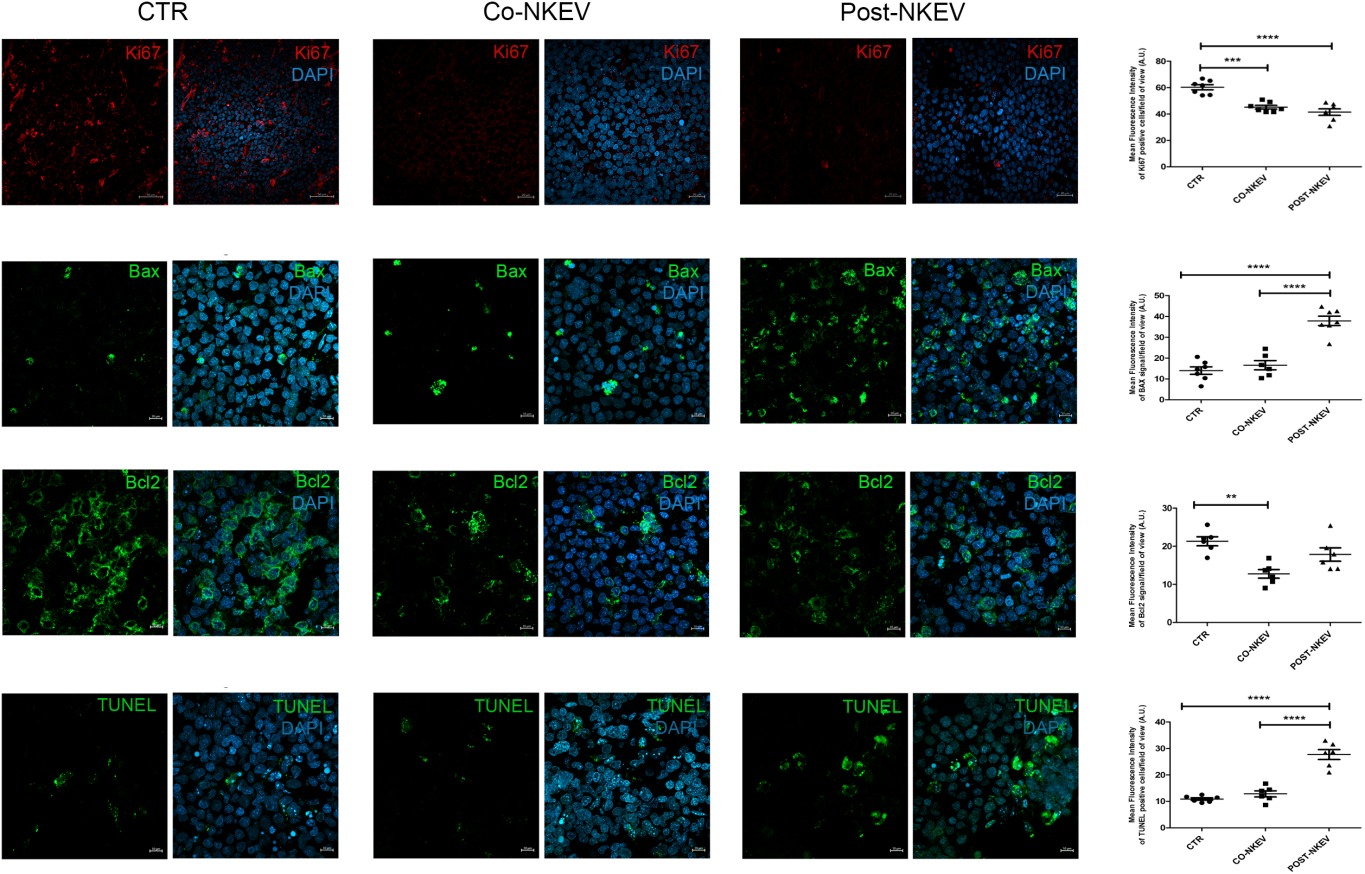
Ex vivo fluorescence analyses of SU-DHL-4 xenografts. Frozen tumor tissues with 10 µm thickness derived from control, Co-NKEV and Post-NKEV groups were stained using Ki67, Bax and Bcl2 Abs, as well as TUNEL staining followed by the respective Alexa Fluor 594 or 488-conjugated secondary Abs and then analyzed by Confocal Laser Scanning Microscopy. CLSM analyses were performed on at least 4 tumor xenografts/group and representative examples are shown. On the right of each panel, the quantification analysis of mean fluorescence intensity is reported. MFI ± SEM of Ki67, Bax, Bcl2 and TUNEL staining signal per field of view (≥300 cells per experimental condition, in 6/7 independent experiments per marker), and plotted in the corresponding graphs, defining final MFI as MFI (region of interest [ROI]) - MFI (background). Bars indicate mean ± SEM, p values were calculated by one-way ANOVA with Tukey’s correction for multiple testing.

We then analysed SU-DHL-4 human follicular B-cell lymphoma xenografts to better characterize the effects of NKEV treatment on tumor tissues. We first performed immunofluorescence staining using anti CD19 and CD38 Abs, markers for neoplastic B cells and haematological tumors in general (**Figure 7**). Our results showed a drastic decrease in the CD19 expression after treatment with both Co-NKEV and Post-NKEV, while CD38 expression was slightly reduced, as also confirmed by the quantification of mean fluorescence intensity. The effects of NKEV treatment were particularly evident in the B-cell lymphoma proliferation rate, as indicated by the decrease in the expression of Ki67 marker (**Figure 8**). Moreover, we evaluated the typical apoptosis-related markers Bax and Bcl2 (**Figure 8**). Intriguingly, pro-apoptotic Bax expression was increased with the Post-NKEV treatment, while the Co-NKEV treatment did not alter Bax expression, compared to the untreated tumors. In contrast, anti-apoptotic Bcl2 expression decreased mainly in the Co-NKEV treatment. TUNEL assay showed an increase in apoptosis in tumors treated with NKEV a week after the B-cell lymphoma injection (Post-NKEV group) (**Figure 8**). Taken together these results indicate that NKEV treatment induced a reduction in the B-cell lymphoma proliferation rate, as well as an induction of apoptosis, with a more pronounced effect obtained with the Post-NKEV treatment.

## Discussion

In this study, we demonstrated that healthy NKEV, both exosomes and microvesicles, have a direct B cell lymphoma antitumor activity inducing apoptosis, both *in vitro* and *in vivo*, and modify the metabolism of tumor mass. In our former work we analyzed by mass spectrometry all the proteins identified in Exo and MV isolated from healthy donors NK cells (18). In this study, we focused our attention in particular in natural killer cell mediated cytotoxicity and apoptosis pathways. Most of the proteins involved in each of these pathways were detected in both types of nanovesicles, Exo and MV. However, *in vitro* experiments demonstrated that the combination of the two nanovesicles have a greater cytotoxic effect against B cell lymphoma. Interestingly, the combination treatment induced an increase in apoptosis (up to 60%) at both 24h and 48h compared to the single use of NKExo and NKMVs (25% and 45%, respectively). Moreover, the treatment with NKEV induced a shift towards apoptotic cell death, thus reducing inflammation risk that could arise from other mechanisms of non-apoptotic cell death (33, 34). Interestingly, among the mechanisms of non-apoptotic cell death, mass spectometry analyses revealed the presence of several proteins involved in necroptosis pathway (**Supplementary Figure 1**). Several conflicting research results support the idea that necroptosis might act as a doubleedged sword in a plethora of pathophysiological conditions. It has been reported that necroptosis can both promote and inhibit tumor growth (35), depending on the type of cancer and whether necroptosis occurs in malignant cells or cells of the tumor microenvironment. Future studies should be performed to investigate whether necroptosis functions as a backup strategy for NKEVs-induced tumor cell death in the event of apoptotic failure. In the KEGG pathway enrichment analysis performed by using DAVID we also found the Neutrophil Extracellular Trap (NET) pathway (**Supplementary Figure 2**). NETs are integral components in the preservation of homeostasis, as evidenced by their involvement in host defense and immune regulation (36). Recent studies suggest that neutrophils actively communicate with other innate and adaptive immune cells, rather than just exerting their phagocytic role (37). Interestingly, NETs play a significant role in the modulation of various immune cell functions (38) and have been implicated extensively in cancer progression, metastatic dissemination, and therapy resistance (39). In light of the crucial role in immune homeostasis exerted by both NK and neutrophils cells, it is reasonable that following their crosstalk an exchange of cellular content can occur and that this material could be stored in the NK extracellular vesicles.

To verify the efficacy of the *in vivo* combination treatment we used SCID mouse xenograft models of human SU-DHL-4 cells. The antitumor effects on tumor growth exerted by NKEV were more pronounced in the group that received NKEV together with the B cell lymphoma injection (Co-NKEV group) (-64%), compared to the Post-NKEV group, that received NKEV a week after B cell lymphoma injection (-44%). These results suggested that the efficacy of NKEV is more pronounced in the early stage of tumor growth by counteracting the initial steps of tumor onset and progression. However, NKEV are still able to tackle tumor growth even when administered a week after tumor injection. The deeper analysis of xenografts with MRI showed homogeneous intensity in both T1 and T2, without any significant morphological alteration (absence of areas of necrosis or hemorrage), confirming a reduction of growth rate, rather than a normal growth, followed by a tumor volume shrinkage due to regions of hypoxia or reduced nutrient support (which is typical of tumors).

The *in vivo* quantification of the MR visible metabolites in xenografts showed a significant increase in the tumor lipid/lactate and in taurine signal in the Co-NKEV group, compared to control and to the Post-NKEV group. Lipid metabolism participates in the regulation of many cellular processes including apoptosis. Lipid molecules promote apoptosis by modulating mitochondrial membrane permeability and activating caspases (31). Taurine has anti-inflammatory, antioxidant, and hypoglycemic effects and possesses antitumor properties, including inhibiting cancer cell proliferation and inducing apoptosis in certain cancers by differential regulating proapoptotic and antiapoptotic proteins (32). Also the trend of increase of glutathione in the Co-NKEV group with respect to control and to the Post-NKEV group detected an altered glutathione antioxidant system, which is associated with multiple forms of programmed cell death in cancer cells (40). *In vivo* MRI/MRS showed previously unexplored NKEV induced metabolic changes in a model of human lymphoma B suggesting taurine and lipid signals as potential biomarkers of NKEV response, providing evidence of altered lipid and redox metabolism following antitumor NKEV treatment.

Moreover, ex vivo analysis by an high resolution (HR)-NMR approach allow the identification of the intratumoral metabolomics profile and define the metabolic effects of NKEV treatment on B-cell lymphoma xenograft model. We found significant changes in the the organic fraction of tissue extracts in the pool of fat acid (FA) and in their degree of unsaturation (UFA). In particular, we detected a significant increase in FA pool and a decrease in UFA and mono-unsaturation (MUFA) both in Co-NKEV and in Post-NKEV groups, as compared to control group. In parallel, the content of saturated fatty acid fraction was significantly different in both treated groups. It has been demonstrated that increased levels of saturated FAs and loss of unsaturated FAs led to cell death in ovarian cancer (41).

Notably, the balance between saturated and unsaturated fatty acids could regulate cancer cell survival and progression, including treatment resistance. Many inhibitors of fatty acid metabolism are currently under development or undergo clinical trial testing (42). Further, in the last years, several studies have identified key differences between saturated fatty acid, MUFA and PUFA metabolism that participate in the maintenance of cancer cells homeostasis including the management of redox stress, energy production (β-oxidation), synthesis of signaling lipids, modification to alter fluidity and permeability of membrane (42).

In many tumor types a relative increase in MUFA-containing glycerophospholipids with corresponding decreases in saturated FAs and PUFAs protect tumor cells from the toxic effects of excess saturated FAs or PUFAs, thereby enhancing cell survival (43, 44). Accordingly, our results demonstrated that NKEV treatment of B-cell lymphoma induces a decrease in UFA in toto, and in MUFA in particular, suggesting that this kind of therapeutic strategy can negatively impact tumor homeostasis, by modifying tumor lipidic metabolism, and could reinforce the action of other agents when combined to form multidrug regimens. In the KEGG pathway enrichment analysis performed by using DAVID we identified several enzymes involve in the biosynthesis of amino acids (**Supplementary Figure 3**). Metabolic reprogramming is a hallmark of the tumor microenvironment and has recently received increased attention. Various nutrients including glucose, lipids, and amino acids, play essential roles in regulating tumorigenesis through acting on both tumor cells and immune cells (45). Among these major nutrients, amino acids play a dominant role in regulating immune cells to impact human tumorigenesis (46). EVs are considered as a native way to deliver bioactive molecules with low cytotoxicity, thus NK-derived extracellular vesicles could transport specific molecules, such as miRNAs, proteins or enzymes associated with the metabolic alteration, to hamper tumor onset and growth.

The apoptotic-induced effect of NKEV was also confirmed by immunohistochemistry and confocal microscopy analyses on SU-DHL-4 xenografts. In fact, we found that NKEV treatment induced a reduction in the B-cell lymphoma proliferation rate, as well as an induction of apoptosis, with a more pronounced effect obtained with the Post-NKEV treatment. The more remarkable apoptotic signals detected in this experiment in the Post-NKEV group, compared to the Co-NKEV, likely depends on the time interval in which these analyses were carried out, that correspond to the end of the experiment (day 28) and to mice sacrifice. On the contrary, *in vivo* MRI analyses were performed during the experiment (starting from day 14 to day 24), and showed a better efficacy in inducing apoptosis with the Co-NKEV treatment. Taking together, these results indicate, however, that both treatments with NKEV are effective against B cell lymphoma, Co-NKEV group seems to be more efficient. The difference between the two treatments could lie in the diverse bioavailability of the nanovesicles in the Co-NKEV group, because in this case tumor cells are exposed to NKEVs in an appropriate ratio, compared to tumors in the Post-NKEV group already organized in a compact mass after one week of growth. Despite the significant advances made in recent times both in conventional treatment approaches, such as surgery, chemotherapy, radiotherapy, and in novel therapeutic strategies, including stem cell therapy, targeted therapy, ablation therapy, nanoparticles, natural antioxidants (47), the desidered results in the fight against cancer have not yet been achieved, mainly due to the tumor adaptation and resistance to therapy and to the toxicity treatment regimens.

In recent years, immunotherapy has been considered one of the best anticancer strategies, including very recent CAR-T and CAR-NK cell therapies (48, 49). Unfortunately, the use of these innovative cell therapies has also encountered several limitations: expensive, time‐consuming process of engineering and expanding T cells, as well as efficacy limitations arising from low major histocompatibility complex (MHC) expression on tumor cells, loss of target antigens, tumor heterogeneity, and immunosuppressive TME (50, 51). Currently, the use of CAR-NK cells seems to be more advantageous than CAR-T. Indeed, in the US Clinical Trials Registry (ClinicalTrials.gov) there are 25 clinical trials of CAR-NK applied to different solid tumors (49), however the use of cell therapy is still invalidated by the immunosuppressive effect of the TME (52).

In the light of these data, promising therapies that can overcome the blockage of the anti-tumor activity of the immune system cells are based on the use of NK cell-derived nanovesicles. Worldwide, many scientists are currently investigating NKEV derived from healthy, immortalized, genetically manipulated or Interleukins-activated NK cells (16–22, 53), thus confirming and strengthening our hypothesis that NK-derived nanovesicles possess a higher therapeutic potential.

Notably, in the presence of an immunocompromised status, the use of NKEV as an anticancer therapy represents a very attractive and promising strategy, similar to the employment of NKEV in supporting NK cell functions. Indeed, both strategies potentially have the goal of removing tumor cells either directly, through the inhibition of tumor growth/induction of cell death, or indirectly, by strengthening the immune system.

All these findings support our hypothesis that in a healthy individual the circulating NKEV can act as sentinels, ready to sustain the immune system in countering cancer onset and in counteracting its growth. The great importance of these immune-nanovesicles, that we have been studying since 2012 (17), relies exactly on the fact that they are constitutively circulating in healthy individuals, derive from untransformed NK cells and are able to directly kill tumor cells. Moreover, NKEV are able to modify tumor lipidic metabolism, negatively affecting tumor homeostasis, thus suggesting that they can be used in future treatment regimens by making tumors more sensitive to combined therapies.

## Data Availability

The datasets presented in this study can be found in online repositories. The names of the repository/repositories and accession number(s) can be found below: https://www.ebi.ac.uk/pride/archive/, PXD014894.

## References

[1] Armitage JO Gascoyne RD Lunning MA Cavalli F. Non-hodgkin lymphoma. Lancet. (2017) 390:298–310. doi: 10.1016/S0140-6736(16)32407-2 28153383

[2] de Leval L Jaffe ES. Lymphoma classification. Cancer J. (2020) 26:176–85. doi: 10.1097/PPO.0000000000000451 32496451

[3] Sehn LH Salles G. Diffuse large B-cell lymphoma. N Engl J Med. (2021) 384:842–58. doi: 10.1056/NEJMra2027612 PMC8377611 33657296

[4] Susanibar-Adaniya S Barta SK. 2021 Update on Diffuse large B cell lymphoma: A review of current data and potential applications on risk stratification and management. Am J Hematol. (2021) 96:617–29. doi: 10.1002/ajh.26151 PMC8172085 33661537

[5] Janeway CA Medzhitov R. Innate immune recognition. Annu Rev Immunol. (2002) 20:197–216. doi: 10.1146/annurev.immunol.20.083001.084359 11861602

[6] Vivier E Tomasello E Baratin M Walzer T Ugolini S. Functions of natural killer cells. Nat Immunol. (2008) 9:503–10. doi: 10.1038/ni1582 18425107

[7] Aptsiauri N Ruiz-Cabello F Garrido F. The transition from HLA-I positive to HLA-I negative primary tumors: the road to escape from T-cell responses. Curr Opin Immunol. (2018) 51:123–32. doi: 10.1016/j.coi.2018.03.006 29567511

[8] Mortezaee K. Immune escape: A critical hallmark in solid tumors. Life Sci. (2020) 258:118110. doi: 10.1016/j.lfs.2020.118110 32698074

[9] Batista IA Quintas ST Melo SA. The interplay of exosomes and NK cells in cancer biology. Cancers. (2021) 13:473. doi: 10.3390/cancers13030473 33530529 PMC7865893

[10] Belting L Hömberg N Przewoznik M Brenner C Riedel T Flatley A. Critical role of the NKG2D receptor for NK cell-mediated control and immune escape of B-cell lymphoma. Eur J Immunol. (2015) 45:2593–601. doi: 10.1002/eji.201445375 26151313

[11] Azoulay T Slouzky I Karmona M Filatov M Hayun M Ofran Y. Compromised activity of natural killer cells in diffuse large b-cell lymphoma is related to lymphoma-induced modification of their surface receptor expression. Cancer Immunol Immunother. (2023) 72:707–18. doi: 10.1007/s00262-022-03284-4 PMC10992952 36048214

[12] Hargadon KM Johnson CE Williams CJ. Immune checkpoint blockade therapy for cancer: An overview of FDA-approved immune checkpoint inhibitors. Int Immunopharmacol. (2018) :62:29–39. doi: 10.1016/j.intimp.2018.06.001 29990692

[13] Fridman WH Zitvogel L Sautès–Fridman C Kroemer G. The immune contexture in cancer prognosis and treatment. Nat Rev Clin Oncol. (2017) 14:717–34. doi: 10.1038/nrclinonc.2017.101 28741618

[14] Théry C Ostrowski M Segura E. Membrane vesicles as conveyors of immune responses. Nat Rev Immunol. (2009) 9:581–93. doi: 10.1038/nri2567 19498381

[15] van Niel G D’Angelo G Raposo G. Shedding light on the cell biology of extracellular vesicles. Nat Rev Mol Cell Biol. (2018) 19:213–28. doi: 10.1038/nrm.2017.125 29339798

[16] Yu D Li Y Wang M Gu J Xu W Cai H. Exosomes as a new frontier of cancer liquid biopsy. Mol Cancer. (2022) 21:56. doi: 10.1186/s12943-022-01509-9 35180868 PMC8855550

[17] Lugini L Cecchetti S Huber V Luciani F Macchia G Spadaro F. Immune surveillance properties of human NK cell-derived exosomes. J Immunol. (2012) 189:2833–42. doi: 10.4049/jimmunol.1101988 22904309

[18] Federici C Shahaj E Cecchetti S Camerini S Casella M Iessi E. Natural-killer-derived extracellular vesicles: immune sensors and interactors. Front Immunol. (2020) 11:262. doi: 10.3389/fimmu.2020.00262 32231660 PMC7082405

[19] Lee EY Park KS Yoon YJ Lee J Moon HG Jang SC. Therapeutic effects of autologous tumor-derived nanovesicles on melanoma growth and metastasis. PloS One. (2012) 7:e33330. doi: 10.1371/journal.pone.0033330 22438914 PMC3305328

[20] Neviani P Wise PM Murtadha M Liu CW Wu CH Jong AY. Natural killer–derived exosomal miR-186 inhibits neuroblastoma growth and immune escape mechanisms. Cancer Res. (2019) 79:1151–64. doi: 10.1158/0008-5472.CAN-18-0779 PMC6428417 30541743

[21] Zhu Q Ling X Yang Y Zhang J Li Q Niu X. Embryonic stem cells-derived exosomes endowed with targeting properties as chemotherapeutics delivery vehicles for glioblastoma therapy. Adv Sci (Weinh). (2019) 6:1801899. doi: 10.1002/advs.201801899 30937268 PMC6425428

[22] Luo H Zhou Y Zhang J Zhang Y Long S Lin X. NK cell-derived exosomes enhance the anti-tumor effects against ovarian cancer by delivering cisplatin and reactivating NK cell functions. Front Immunol. (2023) 13. doi: 10.3389/fimmu.2022.1087689 PMC9892755 36741396

[23] Sherman BT Hao M Qiu J Jiao X Baseler MW Lane HC. DAVID: a web server for functional enrichment analysis and functional annotation of gene lists (2021 update). Nucleic Acids Res. (2022) 50:W216–21. doi: 10.1093/nar/gkac194 PMC9252805 35325185

[24] Geran RI Greenberg N Donald M. Protocols for screening chemical agents and natural products against animal tumors and natural other biological systems. Cancer Chemother Rep. (1972) 3:1–88.

[25] Schiza A Irenaeus S Ortiz-Nieto F Loskog A Tötterman T Sundin A. Evaluation of diffusion-weighted MRI and FDG-PET/CT to assess response to adCD40L treatment in metastatic melanoma patients. Sci Rep. (2019) 9:18069. doi: 10.1038/s41598-019-54438-x 31792256 PMC6889008

[26] Orton MR Messiou C Collins D Morgan VA Tessier J Young H. Diffusion-weighted MR imaging of metastatic abdominal and pelvic tumours is sensitive to early changes induced by a VEGF inhibitor using alternative diffusion attenuation models. Eur Radiol. (2016) 26:1412–9. doi: 10.1007/s00330-015-3933-7 PMC4820470 26253255

[27] Kyriazi S Collins DJ Messiou C Pennert K Davidson RL Giles SL. Metastatic ovarian and primary peritoneal cancer: assessing chemotherapy response with diffusion-weighted MR imaging—Value of histogram analysis of apparent diffusion coefficients. Radiology. (2011) 261:182–92. doi: 10.1148/radiol.11110577 21828186

[28] Canese R Palombelli G Chirico M Sestili P Bagnoli M Canevari S. Integration of MRI and MRS approaches to monitor molecular imaging and metabolomic effects of trabectedin on a preclinical ovarian cancer model. NMR Biomed. (2019) 32. doi: 10.1002/nbm.v32.10 30375088

[29] Canese R Pisanu ME Mezzanzanica D Ricci A Paris L Bagnoli M. Characterisation of in *vivo* ovarian cancer models by quantitative 1 H magnetic resonance spectroscopy and diffusion-weighted imaging. NMR Biomed. (2012) 25:632–42. doi: 10.1002/nbm.v25.4 22020805

[30] Bilgin C Korkmaz B Soylu E Ozturk H Ozturk K. Diffuse large B-cell lymphoma presenting with masses in the pineal and adrenal glands. Clin Case Rep. (2019) 7:577–9. doi: 10.1002/ccr3.2019.7.issue-3 PMC6406144 30899500

[31] Huang C Freter C. Lipid metabolism, apoptosis and cancer therapy. Int J Mol Sci. (2015) 16:924–49. doi: 10.3390/ijms16010924 PMC4307283 25561239

[32] Ma N He F Kawanokuchi J Wang G Yamashita T. Taurine and its anticancer functions: *in vivo* and *in vitro* study. Adv Exp Med Biol. (2022) 1370:121–128. doi: 10.1007/978-3-030-93337-1_11 35882787

[33] Park W Wei S Kim BS Kim B Bae SJ Chae YC. Diversity and complexity of cell death: a historical review. Exp Mol Med. (2023) 55:1573–94. doi: 10.1038/s12276-023-01078-x PMC10474147 37612413

[34] Gao W Wang X Zhou Y Wang X Yu Y. Autophagy, ferroptosis, pyroptosis, and necroptosis in tumor immunotherapy. Signal Transduct Target Ther. (2022) 7:196. doi: 10.1038/s41392-022-01046-3 35725836 PMC9208265

[35] Yan J Wan P Choksi S Liu ZG. Necroptosis and tumor progression. Trends Cancer. (2022) 8:21–7. doi: 10.1016/j.trecan.2021.09.003 PMC8702466 34627742

[36] Islam MM Takeyama N. Role of neutrophil extracellular traps in health and disease pathophysiology: recent insights and advances. Int J Mol Sci. (2023) 24:15805. doi: 10.3390/ijms242115805 37958788 PMC10649138

[37] Mantovani A Cassatella MA Costantini C Jaillon S. Neutrophils in the activation and regulation of innate and adaptive immunity. Nat Rev Immunol. (2011) 11:519–31. doi: 10.1038/nri3024 21785456

[38] Shrestha S Hong CW. Extracellular mechanisms of neutrophils in immune cell crosstalk. Immune Netw. (2023) 23:e38. doi: 10.4110/in.2023.23.e38 37970234 PMC10643328

[39] Zhao J Jin J. Neutrophil extracellular traps: New players in cancer research. Front Immunol. (2022) 13:937565. doi: 10.3389/fimmu.2022.937565 36059520 PMC9437524

[40] Lv H Zhen C Liu J Yang P Hu L Shang P. Unraveling the potential role of glutathione in multiple forms of cell death in cancer therapy. Oxid Med Cell Longev. (2019) 2019:1–16. doi: 10.1155/2019/3150145 PMC6590529 31281572

[41] Zhao G Tan Y Cardenas H Vayngart D Wang Y Huang H. Ovarian cancer cell fate regulation by the dynamics between saturated and unsaturated fatty acids. Proc Natl Acad Sci. (2022) 119. doi: 10.1073/pnas.2203480119 PMC9564215 36197994

[42] Nagarajan SR Butler LM Hoy AJ. The diversity and breadth of cancer cell fatty acid metabolism. Cancer Metab. (2021) 9:2. doi: 10.1186/s40170-020-00237-2 33413672 PMC7791669

[43] Miryaghoubzadeh J Darabi M Madaen K Shaaker M Mehdizadeh A Hajihosseini R. Tissue fatty acid composition in human urothelial carcinoma. Br J Biomed Sci. (2013) 70:1–5. doi: 10.1080/09674845.2013.11669921 23617090

[44] Bandu R Mok HJ Kim KP. Phospholipids as cancer biomarkers: Mass spectrometry-based analysis. Mass Spectrometry Rev. (2018) 37:107–38. doi: 10.1002/mas.21510 27276657

[45] Boroughs LK DeBerardinis RJ. Metabolic pathways promoting cancer cell survival and growth. Nat Cell Biol. (2015) 17:351–9. doi: 10.1038/ncb3124 PMC4939711 25774832

[46] Liu GY Sabatini DM. mTOR at the nexus of nutrition, growth, ageing and disease. Nat Rev Mol Cell Biol. (2020) 21:183–203. doi: 10.1038/s41580-019-0199-y 31937935 PMC7102936

[47] Debela DT Muzazu SG Heraro KD Ndalama MT Mesele BW Haile DC. New approaches and procedures for cancer treatment: Current perspectives. SAGE Open Med. (2021) 9:205031212110343. doi: 10.1177/20503121211034366 PMC8366192 34408877

[48] White LG Goy HE Rose AJ McLellan AD. Controlling cell trafficking: addressing failures in CAR T and NK cell therapy of solid tumours. Cancers. (2022) 14:978. doi: 10.3390/cancers14040978 35205725 PMC8870056

[49] Wang W Liu Y He Z Li L Liu S Jiang M. Breakthrough of solid tumor treatment: CAR-NK immunotherapy. Cell Death Discov. (2024) 10:40. doi: 10.1038/s41420-024-01815-9 38245520 PMC10799930

[50] Algarra I Garcia-Lora A Cabrera T Ruiz-Cabello F Garrido F. The selection of tumor variants with altered expression of classical and nonclassical MHC class I molecules: implications for tumor immune escape. Cancer Immunol Immunother. (2004) 53:904–10. doi: 10.1007/s00262-004-0517-9 PMC11032983 15069585

[51] St. Martin Y Franz JK Agha ME Lazarus HM. Failure of CAR-T cell therapy in relapsed and refractory large cell lymphoma and multiple myeloma: An urgent unmet need. Blood Rev. (2023) 60:101095. doi: 10.1016/j.blre.2023.101095 37173224

[52] Bilotta MT Antignani A Fitzgerald DJ. Managing the TME to improve the efficacy of cancer therapy. Front Immunol. (2022) 13. doi: 10.3389/fimmu.2022.954992 PMC9630343 36341428

[53] Shi Y Chen Y Wang Y Mo D Ai H Zhang J. Therapeutic effect of small extracellular vesicles from cytokine-induced memory-like natural killer cells on solid tumors. J Nanobiotechnol. (2024) 22:447. doi: 10.1186/s12951-024-02676-1 PMC11285333 39075563

