# Supplementary figures and images for: NK cells-derived extracellular vesicles potency in the B cell lymphoma biotherapy
Source: Front Immunol. 2024 Dec 6;15:1503857. doi: 10.3389/fimmu.2024.1503857 (PMC11659271; doi:10.3389/fimmu.2024.1503857)

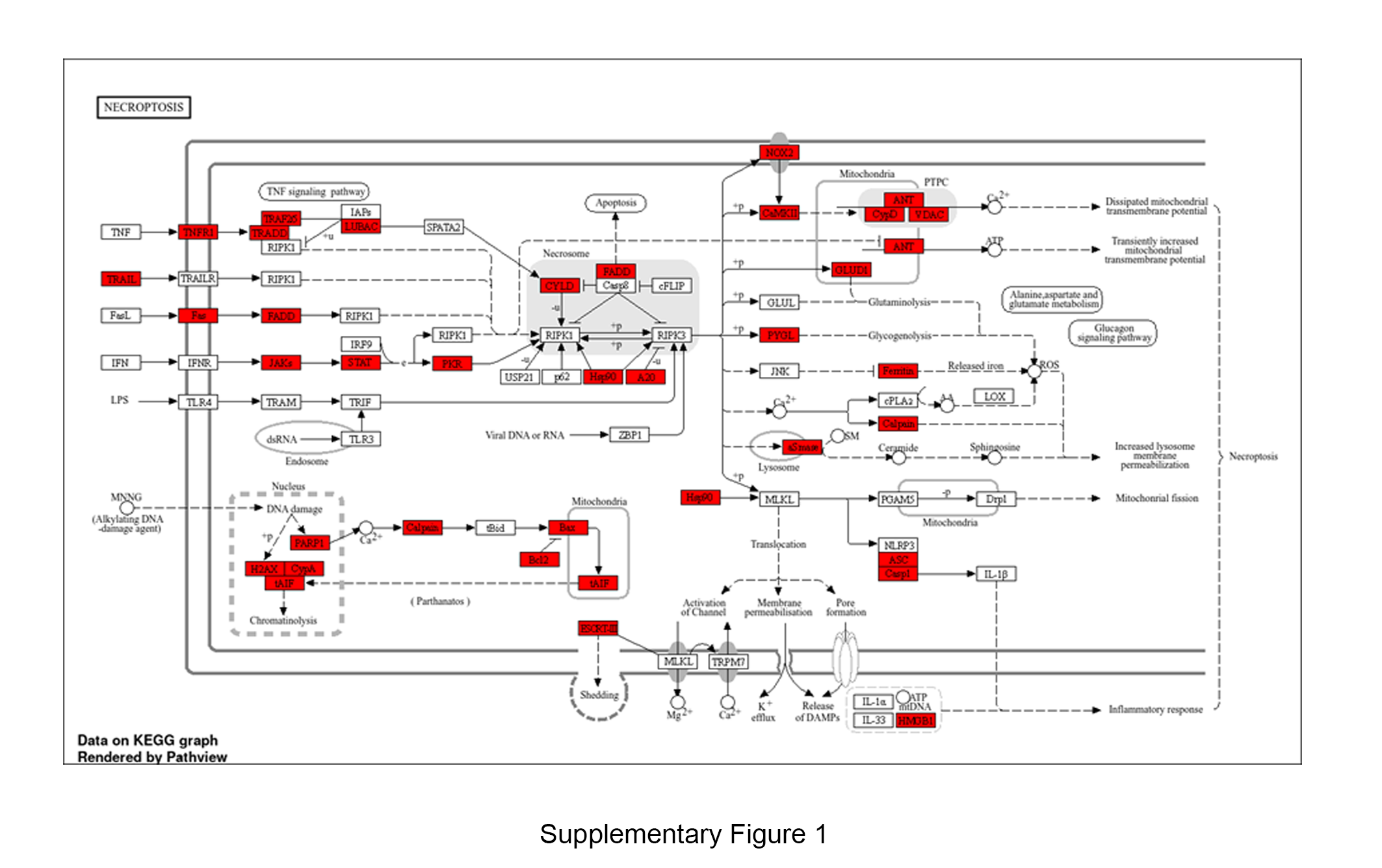

Supplement: Supplementary file 1 [file Image1.tif]

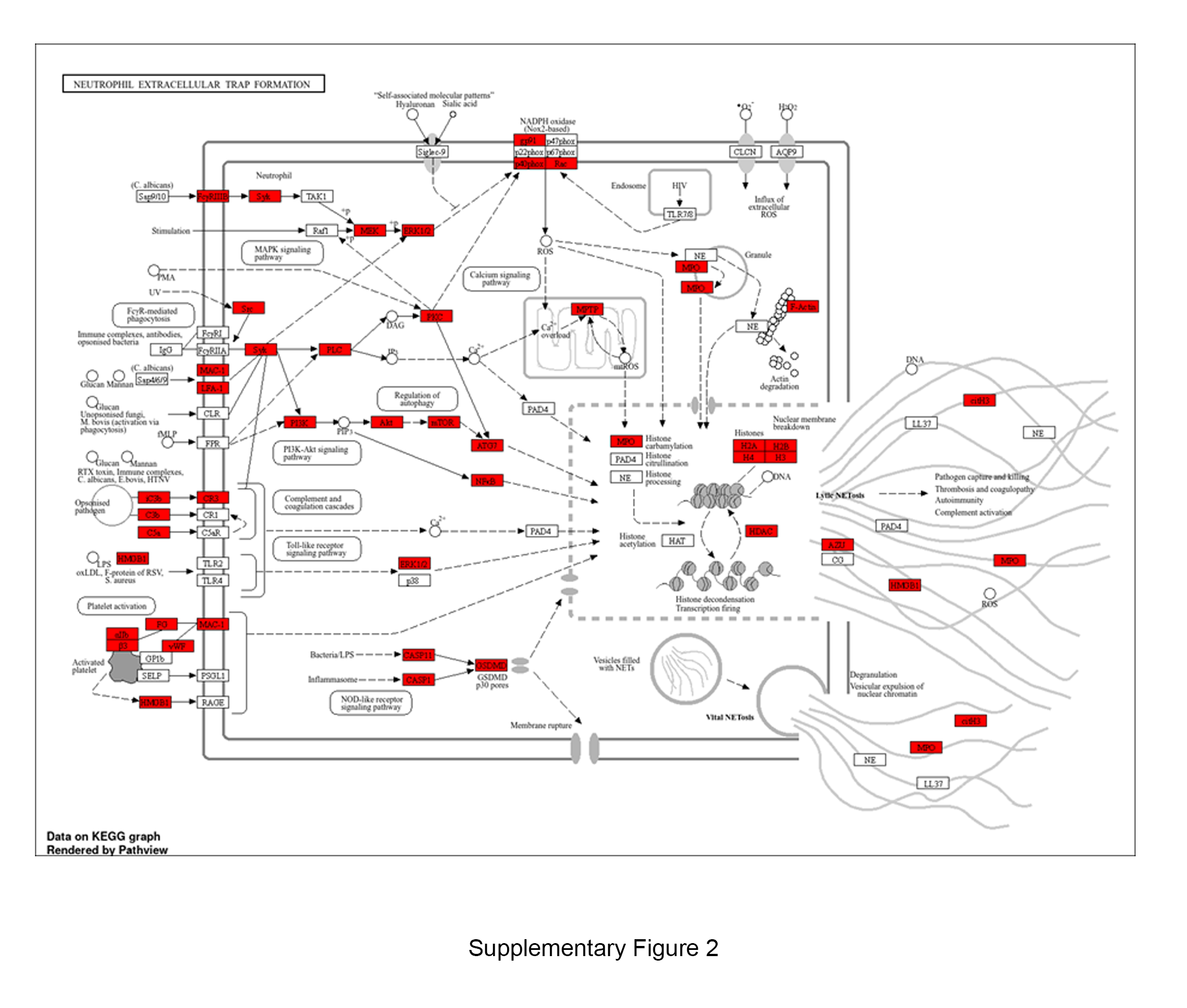

Supplement: Supplementary file 2 [file Image2.tif]

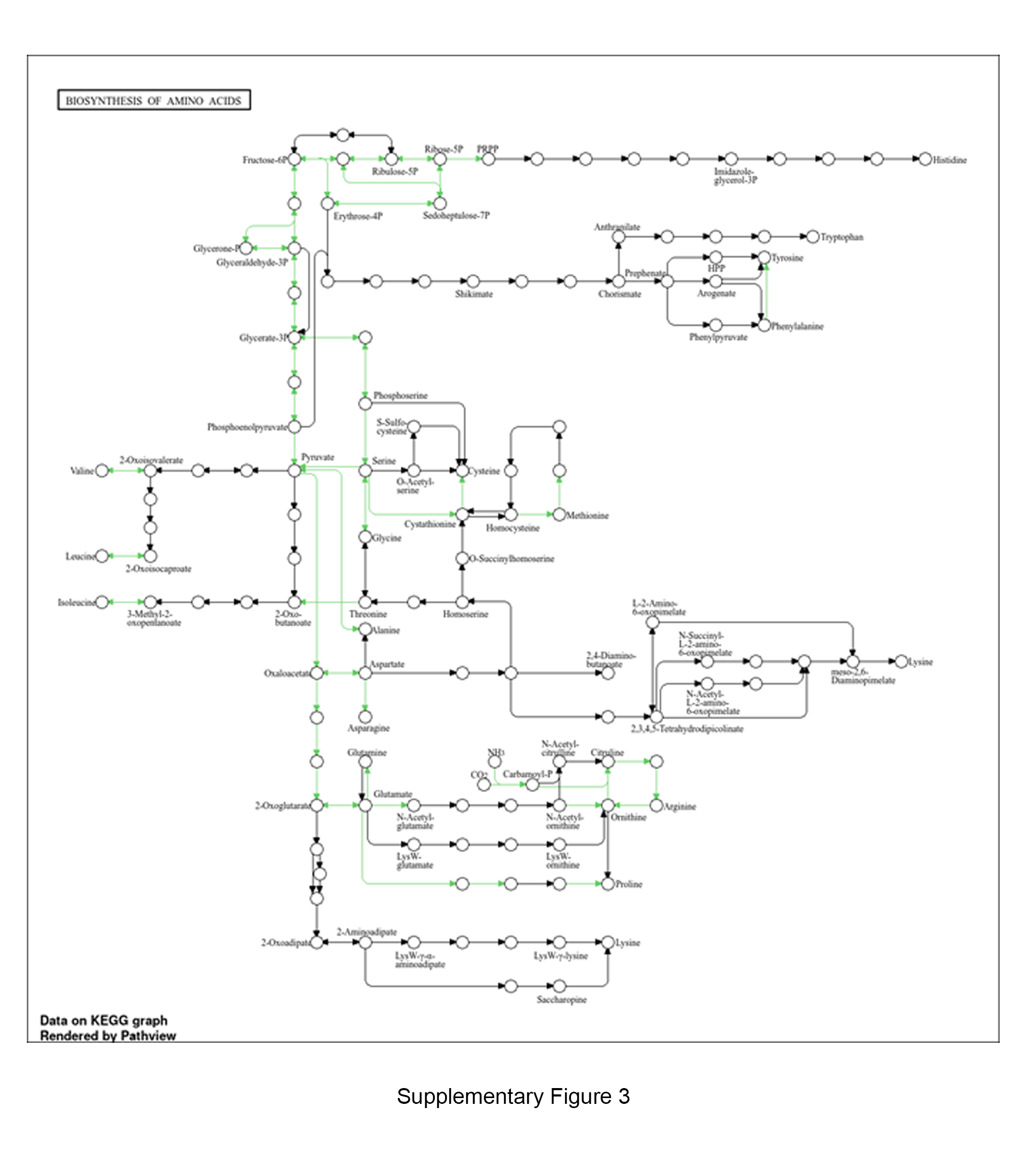

Supplement: Supplementary file 3 [file Image3.tif]
