# Supplementary material for: NK cells-derived extracellular vesicles potency in the B cell lymphoma biotherapy
Source: Front Immunol. 2024 Dec 6;15:1503857. doi: 10.3389/fimmu.2024.1503857 (PMC11659271; doi:10.3389/fimmu.2024.1503857)
Supplement: Supplementary file 4 [file Table1.docx]

**Supplementary table 1. Natural killer mediated cytotoxicity KEGG PATHWAY**

| **Acc Number** | **Protein description** | **Exo** | **MV** |
| --- | --- | --- | --- |
| P43630 | killer cell immunoglobulin like receptor (KIR3DL2) | x |  |
| O43561 | linker for activation of T cells(LAT) | x |  |
| Q07889 | SOS Ras/Rac guanine nucleotide exchange factor 1(SOS1) | x |  |
| P50591 | TNF superfamily member 10(TNFSF10) | x |  |
| O43914 | transmembrane immune signaling adaptor TYROBP(TYROBP) | x |  |
| P10398 | A-Raf proto-oncogene, serine/threonine kinase(ARAF) |  | x |
| P08637 | Fc gamma receptor IIIa(FCGR3A) |  | x |
| P13598 | intercellular adhesion molecule 2(ICAM2) |  | x |
| P17252 | protein kinase C alpha(PRKCA) |  | x |
| Q08209 | protein phosphatase 3 catalytic subunit alpha(PPP3CA) |  | x |
| P63098 | protein phosphatase 3 regulatory subunit B, alpha(PPP3R1) |  | x |
| Q14289 | protein tyrosine kinase 2 beta(PTK2B) |  | x |
| P43405 | spleen associated tyrosine kinase(SYK) |  | x |
| Q9BZW8 | CD244 molecule(CD244) | x | x |
| P20963 | CD247 molecule(CD247) | x | x |
| P09326 | CD48 molecule(CD48) | x | x |
| P25445 | Fas cell surface death receptor(FAS) | x | x |
| P06241 | FYN proto-oncogene, Src family tyrosine kinase(FYN) | x | x |
| P10144 | granzyme B(GZMB) | x | x |
| P62993 | growth factor receptor bound protein 2(GRB2) | x | x |
| P04439 | HLA class I histocompatibility antigen, A alpha chain | x | x |
| P13747 | HLA class I histocompatibility antigen, alpha chain E | x | x |
| P17693 | HLA class I histocompatibility antigen, alpha chain G | x | x |
| P01889 | HLA class I histocompatibility antigen, B alpha chain | x | x |
| P10321 | HLA class I histocompatibility antigen, Cw alpha chain | x | x |
| P20701 | integrin subunit alpha L(ITGAL) | x | x |
| P05107 | integrin subunit beta 2(ITGB2) | x | x |
| P05362 | intercellular adhesion molecule 1(ICAM1) | x | x |
| P43626 | killer cell immunoglobulin like receptor (KIR2DL1) | x | x |
| P26715 | killer cell lectin like receptor C1(KLRC1) | x | x |
| Q13241 | killer cell lectin like receptor D1(KLRD1) | x | x |
| P26718 | killer cell lectin like receptor K1(KLRK1) | x | x |
| P26718 | KLRC4-KLRK1 readthrough(KLRC4-KLRK1) | x | x |
| P01116 | KRAS proto-oncogene, GTPase(KRAS) | x | x |
| P06239 | LCK proto-oncogene, Src family tyrosine kinase(LCK) | x | x |
| Q13094 | lymphocyte cytosolic protein 2(LCP2) | x | x |
| P28482 | mitogen-activated protein kinase 1(MAPK1) | x | x |
| P27361 | mitogen-activated protein kinase 3(MAPK3) | x | x |
| Q02750 | mitogen-activated protein kinase kinase 1(MAP2K1) | x | x |
| P36507 | mitogen-activated protein kinase kinase 2(MAP2K2) | x | x |
| O14931 | natural cytotoxicity triggering receptor 3(NCR3) | x | x |
| P01111 | NRAS proto-oncogene, GTPase(NRAS) | x | x |
| P14222 | perforin 1(PRF1) | x | x |
| O00329 | phosphatidylinositol-4,5-bisphosphate 3-kinase (PIK3CD) | x | x |
| P27986 | phosphoinositide-3-kinase regulatory subunit 1(PIK3R1) | x | x |
| P19174 | phospholipase C gamma 1(PLCG1) | x | x |
| P16885 | phospholipase C gamma 2(PLCG2) | x | x |
| P05771 | protein kinase C beta(PRKCB) | x | x |
| Q06124 | protein tyrosine phosphatase non-receptor type 11(PTPN11) | x | x |
| P29350 | protein tyrosine phosphatase non-receptor type 6(PTPN6) | x | x |
| P63000 | Rac family small GTPase 1(RAC1) | x | x |
| P15153 | Rac family small GTPase 2(RAC2) | x | x |
| O60880 | SH2 domain containing 1A(SH2D1A) | x | x |
| O14796 | SH2 domain containing 1B(SH2D1B) | x | x |
| P29353 | SHC adaptor protein 1(SHC1) | x | x |
| P15498 | vav guanine nucleotide exchange factor 1(VAV1) | x | x |
| P43403 | zeta chain of T cell receptor associated protein kinase 70(ZAP70) | x | x |
