# Supplementary material for: NK cells-derived extracellular vesicles potency in the B cell lymphoma biotherapy
Source: Front Immunol. 2024 Dec 6;15:1503857. doi: 10.3389/fimmu.2024.1503857 (PMC11659271; doi:10.3389/fimmu.2024.1503857)
Supplement: Supplementary file 5 [file Table2.docx]

**Supplementary table 2. Apoptosis KEGG PATHWAY**

| **Acc Number** | **Protein description** | **Exo** | **MV** |
| --- | --- | --- | --- |
| P55212 | caspase 6(CASP6) | x |  |
| P07858 | cathepsin B(CTSB) | x |  |
| Q12933 | TNF receptor associated factor 2(TRAF2) | x |  |
| P19438 | TNF receptor superfamily member 1A(TNFRSF1A) | x |  |
| P50591 | TNF superfamily member 10(TNFSF10) | x |  |
| P31751 | AKT serine/threonine kinase 2(AKT2) |  | x |
| O95831 | apoptosis inducing factor mitochondria associated 1(AIFM1) |  | x |
| O14727 | apoptotic peptidase activating factor 1(APAF1) |  | x |
| Q16611 | BCL2 antagonist/killer 1(BAK1) |  | x |
| P10415 | BCL2 apoptosis regulator(BCL2) |  | x |
| Q07817 | BCL2 like 1(BCL2L1) |  | x |
| Q92851 | caspase 10(CASP10) |  | x |
| Q03252 | lamin B2(LMNB2) |  | x |
| P60709 | actin beta(ACTB) | x | x |
| P31749 | AKT serine/threonine kinase 1(AKT1) | x | x |
| Q07812 | BCL2 associated X, apoptosis regulator(BAX) | x | x |
| P07384 | calpain 1(CAPN1) | x | x |
| P17655 | calpain 2(CAPN2) | x | x |
| P53634 | cathepsin C(CTSC) | x | x |
| P07339 | cathepsin D(CTSD) | x | x |
| P56202 | cathepsin W(CTSW) | x | x |
| O15111 | component of inhibitor of nuclear factor kappa B kinase complex(CHUK) | x | x |
| Q9NR28 | diablo IAP-binding mitochondrial protein(DIABLO) | x | x |
| P05198 | eukaryotic translation initiation factor 2 subunit alpha(EIF2S1) | x | x |
| Q13158 | Fas associated via death domain(FADD) | x | x |
| P25445 | Fas cell surface death receptor(FAS) | x | x |
| P10144 | granzyme B(GZMB) | x | x |
| Q9Y6K9 | inhibitor of nuclear factor kappa B kinase regulatory subunit gamma(IKBKG) | x | x |
| O14920 | inhibitor of nuclear factor kappa B kinase subunit beta(IKBKB) | x | x |
| P01116 | KRAS proto-oncogene, GTPase(KRAS) | x | x |
| P20700 | lamin B1(LMNB1) | x | x |
| P28482 | mitogen-activated protein kinase 1(MAPK1) | x | x |
| P27361 | mitogen-activated protein kinase 3(MAPK3) | x | x |
| Q02750 | mitogen-activated protein kinase kinase 1(MAP2K1) | x | x |
| P36507 | mitogen-activated protein kinase kinase 2(MAP2K2) | x | x |
| Q99683 | mitogen-activated protein kinase kinase kinase 5(MAP3K5) | x | x |
| P01111 | NRAS proto-oncogene, GTPase(NRAS) | x | x |
| P19838 | nuclear factor kappa B subunit 1(NFKB1) | x | x |
| P14222 | perforin 1(PRF1) | x | x |
| O00329 | phosphatidylinositol-4,5-bisphosphate 3-kinase (PIK3CD) | x | x |
| P27986 | phosphoinositide-3-kinase regulatory subunit 1(PIK3R1) | x | x |
| P09874 | poly(ADP-ribose) polymerase 1(PARP1) | x | x |
| Q9UKK3 | poly(ADP-ribose) polymerase family member 4(PARP4) | x | x |
| Q04206 | RELA proto-oncogene, NF-kB subunit(RELA) | x | x |
| Q13813 | spectrin alpha, non-erythrocytic 1(SPTAN1) | x | x |
| Q13077 | TNF receptor associated factor 1(TRAF1) | x | x |
| Q15628 | TNFRSF1A associated via death domain(TRADD) | x | x |
| Q71U36 | tubulin alpha 1a(TUBA1A) | x | x |
| Q9BQE3 | tubulin alpha 1c(TUBA1C) | x | x |
| P68366 | tubulin alpha 4a(TUBA4A) | x | x |
| A6NHL2 | tubulin alpha like 3(TUBAL3) | x | x |
