# Supplementary material for: NK cells-derived extracellular vesicles potency in the B cell lymphoma biotherapy
Source: Front Immunol. 2024 Dec 6;15:1503857. doi: 10.3389/fimmu.2024.1503857 (PMC11659271; doi:10.3389/fimmu.2024.1503857)
Supplement: Supplementary file 6 [file Table3.docx]

**Supplementary Table 3.** Relative quantification **(% metabolite/all metabolites)** of lipid metabolites in Co- NKEV (n=6), in Post-NKEV (n=7) and control tissue extracts (n=5).

| **Lipid Metabolites** | **SALINE** | **Co-NKEV** | **Post-NKEV** |
| --- | --- | --- | --- |
| **Plasmalogen** | 0.07 ± 0.04 | 0.06 ± 0.04 | 0.04 ± 0.02 |
| **Sphingomyelin** | 0.08 ± 0.05 | 0.08 ± 0.04 | 0.07 ± 0.02 |
| **Unsaturated Fatty Acid (UFA)** | **5.64 ± 0.27** | **4.70 ± 0.41** | **3.59 ± 1.56** |
| **Triacylglicerids (TAG)** | 0.45 ± 0.16 | 0.51 ± 0.08 | 0.38 ± 0.24 |
| **Pool of phospholipids (PL)** | 0.59 ± 0.13 | 0.50 ± 0.05 | 0.53 ± 0.08 |
| **pool of phosphatidylcholine (PC) plus Lyso-PC** | 4.28 ± 0.61 | 3.71 ± 0.43 | 3.81 ± 0.70 |
| **phosphatidylethanolamine** | 0.44 ± 0.08 | 0.37 ± 0.06 | 0.39 ± 0.08 |
| **Poly-unsaturated Fatty Acid (PUFA)** | 3.03 ± 0.33 | 2.77 ± 0.21 | 2.75 ± 0.23 |
| **Mono-unsaturated Fatty Acid (MUFA)** | **6.30 ± 0.33** | **5.38 ± 0.48** | **5.35 ± 0.72** |
| **Pool of Fatty Acid (FA)**  determined at 1.6 ppm | **5.22 ± 0.12** | **5.47 ± 0.19** | **5.61 ± 0.19** |
| **Pool of Fatty Acid FA**  determined at 1.9 ppm | 53.24 ± 1.26 | 52.78 ± 1.59 | 53.96 ± 1.12 |
| **Pool of Fatty Acid (FA)**  determined at 0.9 ppm | **12,33 ± 0,87** | **16,12 ± 1,67** | **15,95 ± 2,51** |
| **Total Cholesterol** | 0.97 ± 0.09 | 0.89 ± 0.17 | 0.85 ± 0.11 |

The content of FA was determined at 0.9 ppm (ω-C**H3** of FA), at 1.56 ppm (-C**H2**-COO of FA; β) and at 2.40 ppm (-C**H2**-CH2-COO of FA; β). p <0.05 shown in bold (ANOVA one way test).
