# Supplementary material for: NK cells-derived extracellular vesicles potency in the B cell lymphoma biotherapy
Source: Front Immunol. 2024 Dec 6;15:1503857. doi: 10.3389/fimmu.2024.1503857 (PMC11659271; doi:10.3389/fimmu.2024.1503857)
Supplement: Supplementary file 7 [file Table4.docx]

**Supplementary Table 4.** Absolute quantification (**nanomoles/mg tissues)** of aqueous metabolites in Co- NKEV (n=6), in Post-NKEV (n=7) and control tissue extracts (n=5).

| **Aqueous Metabolites**  **(nanomoles/mg tissues)** | **SALINE** | **Co-NKEV** | **Post-NKEV** |
| --- | --- | --- | --- |
| **NADP** | 217.1±81.7 | 244.8±96.6 | 114.3±144.8 |
| **NAD** | 238.2±75.5 | 270.3±90.7 | 114.5±145.6 |
| **Nicotinammide** | 86.9±104.0 | 72.5±15.3 | 17.1±31.6 |
| **AMP** | 636.9±383.5 | 778.9±204.9 | 795.5±346.6 |
| **ATP+ADP** | 1886.9±1013.7 | 2082.6±847.9 | 2756.1±916.2 |
| **Formic acid** | 1623.1±1094.4 | 3032.7±1829.8 | 2487.9±582.5 |
| **Histidine** | 158.2±87.7 | 184.8±57.7 | 231.9±96.5 |
| **Tyrosine** | 429.6±213.4 | 513.9±166.4 | 622.7±319.3 |
| **Fumaric acid** | 16.6±14.8 | 23.5±32.4 | 4.7±16.0 |
| **Glucose** | 392.0±321.4 | 729.6±377.6 | 441.4±235.2 |
| **Lactic acid** | 4714.0±2264.5 | 5236.1±1684.1 | 6857.4±964.1 |
| **Taurine (Tau)** | 15828.6±13283.4 | 14446.9±11780.0 | 20834.0±11136.9 |
| **Creatine** | 582.3±260.1 | 724.0±252.8 | 822.1±348.4 |
| **glutathione** | 2499.6±1489.3 | 2192.4±704.2 | 2983.3±1016.7 |
| **L-Glutamine** | 1240.3±603.8 | 1619.1±552.1 | 1762.9±501.9 |
| **Succinic acid** | 281.4±123.9 | 328.4±120.3 | 410.9±153.1 |
| **Glutamic acid** | 6827.2±2915.8 | 7905.3±2550.7 | 8966.7±3186.1 |
| **N-acetyl compounds** | 2960.4±1344.8 | 3456.8±1133.3 | 3893.1±1335.6 |
| **Acetic acid** | 648.8±226.2 | 931.7±325.9 | 1081.6±436.5 |
| **Alanine** | 6659.6±3452.6 | 6730.6±2310.4 | 8611.5±2645.9 |
| **3-hydroxy-butyric acid** | 330.2±175.1 | 598.35±359.11 | 495.4±173.6 |
| **Isoleucine** | 451.0±214.7 | 587.8±172.9 | 649.1±221.5 |
| **Valine** | 563.7±286.7 | 813.6±258.2 | 853.8±301.1 |
